# Occurrence and Risk Assessment of Antimicrobials and Resistant Bacteria in Treated Sewage Effluents in South Brazil

**DOI:** 10.3390/antibiotics14080836

**Published:** 2025-08-18

**Authors:** Keite da Silva Nogueira, Ana Paula de Oliveira Tomaz, Gabrielly Cristina Kubis, Raizza Zorman Marques, Nicole Geraldine de Paula Marques Witt, Aliny Lucia Borges Borba, Bárbara Zanicotti Leite, Marcelo Pedrosa Gomes

**Affiliations:** 1 Departamento de Patologia Básica, Setor de Ciências Biológicas, Universidade Federal do Paraná, Avenida Coronel Francisco H. dos Santos, 100, Centro Politécnico Jardim das Américas, Curitiba 81531-980, PR, Brazil; keite.nogueira@ufpr.br; 2 Laboratório de Bacteriologia, Complexo Hospital de Clínicas, Universidade Federal do Paraná, Rua Padre Camargo, 280, Curitiba 80060-240, PR, Brazil; 3 Laboratório de Fisiologia de Plantas sob Estresse, Departamento de Botânica, Setor de Ciências Biológicas, Universidade Federal do Paraná, Avenida Coronel Francisco H. dos Santos, 100, Centro Politécnico Jardim das Américas, Curitiba 81531-980, PR, Brazil; 4 Companhia de Saneamento do Paraná (SANEPAR)—Sede Administrativa, Rua Engenheiros Rebouças, 1376, Curitiba 80215-900, PR, Brazil

**Keywords:** wastewater treatment plants, resistant bacteria, urban effluents, ecotoxicology, water pollution

## Abstract

**Background/Objectives:** The increasing presence of antimicrobial residues and antibiotic-resistant bacteria (ARB) in effluents from wastewater treatment plants (WWTP) has become a critical concern for environmental and public health. This study aimed to investigate the occurrence, concentrations, and ecological risks of commonly used antimicrobials as well as the prevalence of clinically relevant ARB in treated effluents. **Methods:** A five-month monitoring campaign was conducted at a major WWTP in Curitiba, Brazil. Thirteen antibiotics were quantified using LC-MS/MS, resistant bacteria were identified via phenotypic profiling, and ecotoxicological assays were performed with *Desmodesmus subspicatus*. Risk assessments included hazard quotient (HQ) calculations for ecotoxicity and resistance selection as well as multivariate and correlation analyses. **Results:** All antibiotics were consistently detected over five months, with total concentrations ranging from 1730 to 2840 ng L^−1^. Clinically relevant ARB (*Escherichia coli*, *Klebsiella pneumoniae*, and *Enterobacter cloacae*) resistant to high-priority antibiotics were also isolated. Ecotoxicological tests showed moderate growth inhibition only in undiluted effluent. HQ values for ecotoxicity were <1, but HQ for resistance selection exceeded 1 for all compounds. Multivariate analyses showed strong associations between fluoroquinolone and macrolide concentrations and ARB detection. **Conclusions:** Although WWTPs reduce pollutant loads, conventional processes may not fully eliminate antimicrobials and ARB, highlighting the need for advanced treatments. Culture-based detection may have underestimated the resistance diversity. These findings support the integration of resistance-based discharge thresholds into regulations, and provide a replicable model for AMR surveillance in tropical urban systems.

## 1. Introduction

Antimicrobial resistance (AMR) is a major global health concern, recognized by the World Health Organization as one of the top ten threats to public health and development. Although primarily associated with clinical settings, increasing evidence indicates that aquatic ecosystems play a critical role in the spread and environmental persistence of resistance genes and antibiotic-resistant bacteria (ARB) [1]. Among the anthropogenic contributors to this phenomenon, urban wastewater treatment plants (WWTPs) are of particular interest because of their central role in collecting and processing effluents containing antimicrobial residues, ARB, and resistance genes from diverse sources including households, hospitals, industry, and agriculture [2]. As urban populations grow and antibiotic use increases, WWTPs increasingly receive high loads of antimicrobials and resistant microorganisms. Although these systems play a critical role in pollutant reduction, residual antimicrobial compounds and resistant bacteria may still be discharged into aquatic ecosystems because of the limited removal efficiency of conventional processes [3,4,5].

In urban systems, antimicrobials used in human and veterinary medicine, agriculture, and aquaculture often remain unmetabolized, or are only partially transformed. These compounds enter WWTPs, and owing to the limitations of conventional treatments, they may be released into surface waters in their active forms [2,3]. Antibiotics are extensively used in both human and veterinary medicine in the region studied. Additionally, nearby agricultural and aquaculture activities may contribute to antimicrobial inputs, indirectly influencing effluent composition. Rather than serving as sources of AMR, WWTPs should be understood as critical barriers that reduce contaminant loads, and as strategic monitoring points to mitigate environmental antimicrobial resistance. However, standard physicochemical and biological treatments are not specifically designed to remove trace levels of pharmaceuticals or biological agents, such as ARB and antimicrobial resistance genes (ARGs), that may persist in treated effluents [1].

The presence of residual antimicrobials and resistance determinants in effluents discharged into rivers, reservoirs, and wetlands may facilitate selective pressure, horizontal gene transfer, and the downstream dissemination of resistance [6]. From an epidemiological perspective, these findings underscore the role of WWTPs as strategic monitoring points for antimicrobial resistance in the environment rather than as sources of its emergence [7]. Few studies in Latin America have simultaneously integrated chemical quantification, resistance profiling, and risk modeling in real-world WWTP systems despite growing concerns. Most monitoring efforts have focused on basic chemical and microbiological parameters, without addressing pharmaceutical or resistance risks. Furthermore, only a few studies have combined chemical occurrence, microbiological analysis, and quantitative risk assessments in real-world systems.

In addition to the propagation of resistance, several antimicrobials have been shown to be toxic to nontarget aquatic organisms [8,9]. Although individual concentrations in WWTP effluents are often below ecotoxicological thresholds, their chronic exposure, persistence, and synergistic effects may lead to sublethal or cumulative impacts [1]. Recent global health events, such as the COVID-19 pandemic, have intensified concerns regarding antimicrobial overuse and environmental exposure. In Brazil, the consumption of azithromycin alone has increased by over 71% in 2021 compared to the previous year, driven by its off-label use in COVID-19 treatment protocols [10]. Moreover, interactions between antimicrobials and environmental matrices may increase toxicity, highlighting the importance of mixture-based assessment [11].

This study aimed to (i) evaluate the occurrence and concentrations of selected antibiotics in the treated effluents of two WWTPs in Curitiba, Southern Brazil, (ii) identify the presence of clinically relevant antibiotic-resistant bacteria, and (iii) assess ecological and resistance-selection risks using both deterministic and probabilistic models. To our knowledge, this is the first study in South America to jointly employ LC-MS/MS antibiotic quantification, phenotypic resistance profiling, ecotoxicological bioassays, and probabilistic resistance risk modeling to evaluate the treated wastewater effluents. Our findings contribute to the understanding of the limitations of conventional WWTPs in addressing antimicrobial contaminants, and provide evidence to support the implementation of complementary treatment technologies and monitoring frameworks in Brazil.

## 2. Results

### 2.1. Physicochemical Parameters

The effluent pH remained stable throughout the study period, ranging from 6.3 to 7.2. Temperature varied seasonally, with the lowest value recorded in June (18.4 °C) and the highest in January (26.5 °C). Conductivity and TSS fluctuated between 320 and 435 µS cm^−1^ and 85–144 mg L^−1^, respectively, with peak values in March (Figure S1). Despite natural variability, all parameters remained within Brazilian regulatory limits (CONAMA Resolution No. 430/2011).

### 2.2. Antimicrobial Concentrations

**Figure 1:**
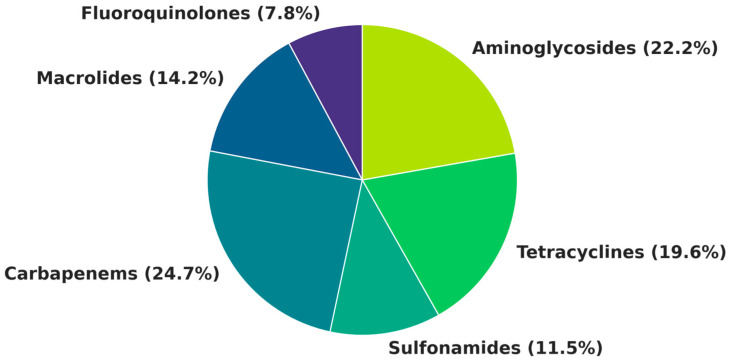
Relative contribution of each antibiotic class to the total concentration detected in treated effluent samples.

Thirteen antimicrobials were consistently detected in the treated effluent of the WWTP (Figure 1). The total concentrations ranged from 1730 to 3290 ng L^−1^ across months, with a mean of 2351 ± 572 ng L^−1^. Considering the mean total concentration, carbapenems accounted for the largest proportion (24.7%, 358 ng L^−1^), followed by aminoglycosides (22.2%, 323 ng L^−1^) and tetracyclines (19.6%, 284 ng L^−1^). Macrolides, sulfonamides, and fluoroquinolones contributed to 14% (206 ng L^−1^), 11.5% (167 ng L^−1^), and 8% (114 ng L^−1^), respectively (Figure 1; Table 1). All the compounds were detected monthly at levels above the analytical limit.

**Figure 2:**
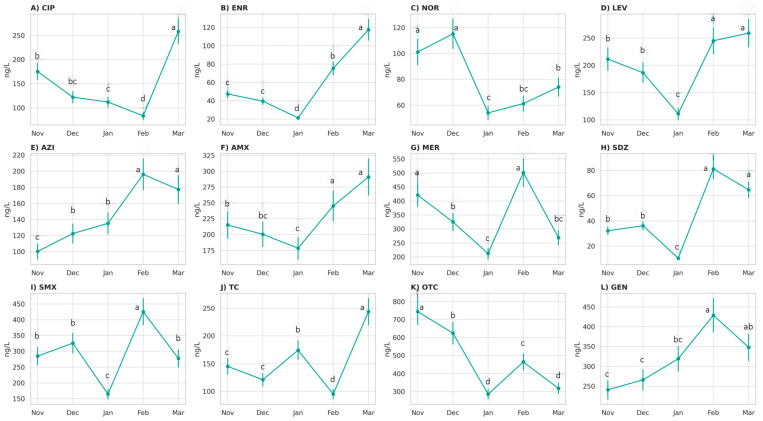
Concentrations of ciprofloxacin (**A**), enrofloxacin (**B**), norfloxacin (**C**), levofloxacin (**D**), azithromycin (**E**), amoxicillin (**F**), meropenem (**G**), sulfamethoxazole (**H**), sulfadiazine (**I**), tetracycline (**J**), oxytetracycline (**K**), and gentamicin (**L**) found in effluents from a WWTP in Curitiba, Brazil, from November 2022 to March 2023. Values represent mean ± standard deviation (*n* = 4). Different lowercase letters indicate statistically significant differences between months (*p* < 0.05, Tukey’s HSD test).

Temporal variability was evident for several compounds (*p* < 0.05; Table S3). CIP and ENR concentrations peaked in March 2023 (Figure 2A,B). NOR concentrations were the highest in December 2022 (Figure 2C), whereas LEV concentrations were lower in January 2023 (Figure 2D). AZI peaked in February 2023 (Figure 2E). AMX concentrations increased from January 2023, peaking in February and March 2023 (Figure 2F). MER concentrations showed a notable decrease from December 2022 (Figure 2H), whereas SDZ and SMX peaked in February 2023 (Figure 2I). TC reached its highest concentration in March 2023 (Figure 2J), whereas OTC was the most concentrated in November 2022 (Figure 2K). GEN concentrations increased and peaked in February 2023 (Figure 2L).

### 2.3. Ecotoxicological Effects on D. subspicatus

Except for the undiluted effluent (100%), the mean algal density remained above 850,000 cells mL^−1^ in all treatments and the optical density curves confirmed exponential growth over 72 h (Table S1). No morphological alterations were observed by optical microscopy.

### 2.4. Toxicological Risk Assessment

The hazard quotients for ecotoxicological risk (HQ_ecotox_) were below one for all compounds in relation to the majority of the species included in the assessment (Table S2), indicating a low immediate risk from individual substances (Table 2). However, the mixture toxicity estimates revealed a more concerning scenario. The multisubstance potentially affected fraction (msPAF_Total_) reached 0.49 for acute and 0.57 for chronic scenarios, indicating that up to 50–57% of aquatic species may be affected by the combined presence of antibiotics in the effluent, thereby posing a significant ecological risk to biodiversity. Ciprofloxacin was the dominant contributor to mixture toxicity in both the acute (92.37%) and chronic (78.19%) assessments. Other relevant contributors included meropenem (7.75% acute and 13.77% chronic), azithromycin (2.10% acute and 11.93% chronic), and amoxicillin (1.88% acute and 4.94% chronic). All other compounds contributed to less than 3% of the total mixture toxicity in both scenarios.

Probabilistic risk modeling based on log-normal distributions of MEC/PNEC_res. sel._ ratios revealed that all assessed antibiotics exhibited hazard quotients for resistance selection (HQ_res.sel._) exceeded a risk threshold of 1 (Table 3). The highest values were observed for MER (5601), CIP (2033), AMX (1043), and ENR (1019). The probability of exceeding the predicted no-effect concentration for resistance selection (Φ_i_) was 1.0, indicating a near-certain likelihood that each substance posed a resistance selection risk. Consequently, the cumulative risk (Φtotal) was 1.0, representing a worst-case scenario in which at least one antibiotic consistently exceeded its PNEC_res.sel._ threshold for the mixture (Table 3). 

### 2.5. Detection of Antimicrobial-Resistant Bacteria

Antimicrobial-resistant bacteria were isolated from all samples evaluated (Table 4 and Table S3), and the resistance was detected based solely on phenotipic methods. *Enterobacterales* producing extended-spectrum β-lactamases (ESBLs) and carbapenemases were isolated monthly. ESBL-producing isolates were resistant to penicillin and cephalosporins, while carbapenemase-producing isolates were resistant to penicillin, cephalosporins, and carbapenems. In addition, many isolates were resistant to ciprofloxacin, amikacin, gentamicin, and sulfamethoxazole-trimethoprim. The most frequent species were *Escherichia coli* and the *Klebsiella pneumoniae* complex, followed by the *Enterobacter cloacae* complex (Table 4). Carbapenem-resistant strains of *Acinetobacter baumannii* and *Pseudomonas aeruginosa* were not isolated in the present study. Other *Pseudomonas* sp. and *Acinetobacter* sp., in addition to *Aeromonas* sp., were isolated, as shown in the Supplemental Materials (Table S3).

### 2.6. Correlation and Multivariate Analysis

**Figure 3:**
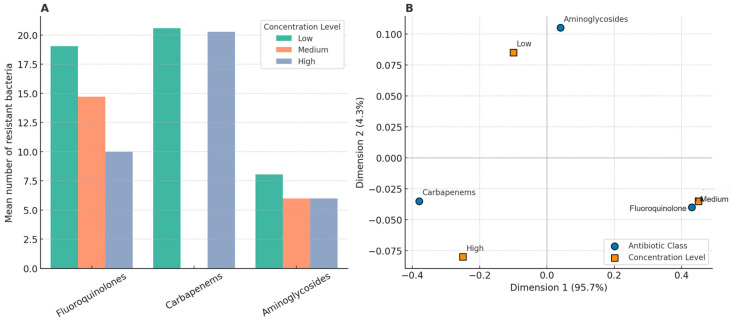
Relationship between antibiotic concentration and the number of antibiotic-resistant bacteria of medical interest. (**A**) The mean number of resistant bacteria observed per antibiotic class and concentration. Only antibiotic classes with corresponding bacterial resistance data are shown. (**B**) Correspondence analysis showing the association between antibiotic classes and concentration categories. The first two dimensions account for 100% of the variance.

The frequency and type of antibiotic-resistant bacteria vary across months and classes. When grouped by pharmacological class and concentration levels, the number of resistant isolates was the highest for macrolides and carbapenems under high concentration conditions and for fluoroquinolones at medium levels (Figure 3). Pearson’s correlation analysis did not reveal any statistically significant linear association between antibiotic concentration (as a continuous variable) and resistance frequency for any class (CIP, *p* = 0.37; ENR, *p* = 0.44; AZI, *p* = 0.53; AMX, *p* = 0.62; MER, *p* = 0.41). However, correspondence analysis revealed a distinct pattern. No clear association was observed for sulfonamides, tetracyclines, or aminoglycosides, which were dispersed across the concentration levels or absent.

## 3. Discussion

Our findings provide strong evidence that despite their crucial role in pollutant mitigation, WWTPs may not fully remove all micropollutants, and thus, treated domestic effluents can still contain residual antimicrobials and ARB. Although physicochemical parameters remained within regulatory limits throughout the study, the consistent detection of all 13 target antimicrobials and the isolation of clinically relevant ARB across five months highlight the limitations of conventional treatment technologies in achieving complete removal of certain contaminants. Although influent samples were not analyzed in this study, our aim was not to evaluate treatment performance, but rather to characterize the potential environmental and public health risks associated with residual antimicrobials and resistant bacteria in treated effluents. Previous studies have reported variable removal efficiencies of antibiotics in WWTP, including negative or incomplete removal of compounds such as CIP, AZI, SMX, and TET (from −71.6% to 56.3%) [12,13,14]. These findings support the relevance of assessing the treated effluents as a critical environmental matrix, particularly in the context of antimicrobial resistance monitoring. Rather than positioning WWTPs as sources of AMR, these findings reinforce their importance as strategic monitoring and intervention points for broader efforts to mitigate antimicrobial resistance in urban aquatic environments.

Source attribution between human and veterinary inputs remains uncertain because ENR is exclusively used in veterinary medicine, whereas all other compounds have been approved for use in human and animal therapies. Therefore, it was not possible to quantitatively estimate the relative contributions of each source based solely on the detected concentrations. Interestingly, the median concentration of ENR, which is exclusively used in veterinary medicine, was lower than most other antimicrobials detected. This finding supports the notion that antimicrobial resistance cannot be attributed solely to the veterinary sector. Instead, it underscores the complex, multi-sectoral nature of AMR emergence, which involves human medicine, environmental dissemination, and agricultural use. Therefore, a comprehensive and coordinated approach, such as the One Health framework, is essential to fully understand and address the origins and spread of antimicrobial resistance [15,16].

The persistent presence of fluoroquinolones, β-lactams,, and tetracyclines reflects their widespread consumption and partial removal from WWTPs, as previously reported in Brazil and other countries [17,18,19,20]. Recent studies have reported antimicrobial concentrations in WWTP effluents reaching 3403 ng L^−1^ for CIP, 2900 ng L^−1^ for ENR, 9347 ng L^−1^ for NOR, 980 ng L^−1^ for AZI, 1600 ng L^−1^ for AMX, 1309 ng L^−1^ for SMX, 1536 ng L^−1^ for TET, 2014 ng L^−1^ for OTC, and up to 67.9 ng L^−1^ for MER [19,20]. In our study, the observed concentrations were lower but still environmentally relevant, with fluoroquinolones and macrolides frequently exceeding resistance selection thresholds (e.g., CIP = 302.6 ng L^−1^; AZI = 211.1 ng L^−1^). Seasonal variations in antibiotic concentration have been linked to regional consumption patterns, illness prevalence,, and flow variability [21,22,23]. The observed seasonal fluctuations, particularly for fluoroquinolones, macrolides, and β-lactams, likely reflect increased antibiotic consumption during warmer months due to seasonal infections and self-medication practices. The detection of high concentrations of meropenem, a last-resort antibiotic, may indicate its excessive clinical use or incomplete removal in the current treatment configuration. In the case of azithromycin, the elevated levels in January suggest a lingering effect of COVID-19-related off-label use. These differences in concentrations compared to other studies may also be attributed to variations in sampling strategy (e.g., grab vs. composite sampling), dilution effects in the receiving environment, or differences in removal efficiency across WWTPs. In this study, samples were collected directly from the outflow of a single WWTP, limiting comparability to riverine systems that integrate multiple pollution sources. Nonetheless, the detection of priority antimicrobials at levels surpassing PNEC values highlights the need for enhanced treatment processes and regulatory frameworks.

Although individual HQ_ecotox_ values were below 1 for all antibiotics across the majority of tested species, suggesting a low risk from isolated exposure, the mixture toxicity analysis revealed a more critical picture. Under acute and chronic exposure scenarios, the cumulative toxicity of the antibiotic mixture reached 0.49 under acute and 0.57 under chronic exposure scenarios, indicating that approximately half of the aquatic species may be at risk owing to combined effects. These results highlight the limitations of evaluating pharmaceuticals in isolation, and emphasize the need to consider mixture-level interactions in environmental risk assessments. Ciprofloxacin has emerged as the primary driver of mixture toxicity, contributing >90% under acute conditions and >78% under chronic exposure, followed by meropenem, azithromycin, and amoxicillin. These findings highlight the disproportionate ecological influence of a few potent antibiotics, particularly fluoroquinolones and β-lactams, on the ecotoxicological profile of treated effluents. This evidence also underscores an important gap in current regulatory frameworks that typically rely on single-compound thresholds for environmental safety assessments. However, the demonstrated mixture toxicity and selection pressure suggest that such approaches may underestimate the real-world risks. Incorporating mixture-level assessments into legislation and risk-management strategies would better reflect the cumulative ecological burden posed by the simultaneous exposure to multiple pharmaceuticals, even at low concentrations. This adjustment is particularly relevant for urban wastewater systems where compound interactions are unavoidable. Hence, regulatory frameworks should prioritize not only individual compound thresholds but also incorporate mixture-based assessments to better protect aquatic biodiversity.

While no acute effects were observed in the *D. subspicatus* bioassays and algal growth was maintained, such tests may not capture the sublethal or chronic effects associated with complex contaminant mixtures or AMR propagation. Therefore, it is essential to complement traditional ecotoxicity tests using microbiological and genomic tools to achieve holistic risk assessment. Although individual antimicrobial concentrations were below ecotoxicological thresholds, probabilistic modeling revealed that HQ_res. sel._ values for all compounds far exceeded the risk threshold of 1 [24], reaching values of up to 5601, indicating a high likelihood of resistance selection even when direct toxicity was negligible. Meropenem, ciprofloxacin, amoxicillin, and enrofloxacin had the highest values. The probability of resistance selection (Φ_i_) was 1.0, and the cumulative probability (Φ_total_) for the mixture was 1.0, indicating a worst-case scenario in which at least one compound consistently exceeded its resistance selection threshold. These results reinforce the notion that conventional toxicity-based evaluations may underestimate real environmental risks and that resistance selection pressure must be addressed as a critical ecological concern.

Indeed, ARB were detected throughout the five-month evaluation, and the isolation of *E. coli* and *K. pneumoniae* from all samples, including ESBL- and carbapenemase-producing isolates resistant to critically important antimicrobials (e.g., MER, CEF, and CIP), raises serious concerns regarding public and environmental health. It is important to note that resistance detection in this study relied exclusively on phenotypic methods, which may have underestimated the overall diversity of ARB and excluded viable but non-culturable (VBNC) organisms or those carrying resistance genes not expressed under laboratory conditions. Thus, the recovered resistant strains likely represent only a subset of the total resistance potential of the effluent. Future studies employing metagenomics or quantitative PCR could provide a more comprehensive view of resistance gene abundance, mobility, and microbial community structure, thereby complementing the phenotypic approach used in this study. These microorganisms are part of a critical group listed on the World Health Organization Priority Pathogen List [25]. Similar patterns have been reported in studies conducted in Brazilian hospital-impacted rivers [1,18], but few studies have simultaneously integrated resistance profiles, antimicrobial quantification, and resistance risk modeling in the Latin American context. By adopting a holistic and probabilistic approach, this study bridges this gap and enhances our understanding of the AMR dissemination pathways through effluent treatment.

Despite the lack of significant linear correlations in Pearson’s analysis, correspondence analysis provided insights into potential class-level risks. The clustering of macrolides and carbapenems with high concentrations in the ordination space supports the hypothesis that these classes may exert a greater selective pressure under environmental exposure. This pattern may be attributed to several factors, including their relatively high environmental stability, frequent clinical use, and the presence of well-characterized resistance mechanisms (e.g., macrolide efflux pumps and carbapenemase enzymes) that facilitate selection and persistence. Moreover, macrolides are known to accumulate in biofilms, whereas carbapenems, despite being considered last-resort drugs, have shown incomplete removal in WWTPs, thereby contributing to their environmental impact [26]. This reinforces the inadequacy of relying solely on linear models to assess AMR risk, and emphasizes the value of multivariate ecological tools. The lack of clear associations between aminoglycosides and sulfonamides may stem from either their low persistence or rapid degradation in environmental matrices. From an epidemiological perspective, these findings reaffirm the role of WWTPs as surveillance nodes for antimicrobial resistance in aquatic environments, rather than sources. Effluents containing resistant bacteria and sub-inhibitory concentrations of antibiotics may reach downstream communities, fisheries, and agricultural areas, thereby creating indirect exposure routes. Thus, WWTPs should be leveraged as environmental sentinel systems for AMR tracking and mitigation within the One Health Framework.

This study is also methodologically innovative. To our knowledge, this is the first study in South America to integrate LC-MS/MS quantification, phenotypic resistance profiling, ecotoxicological bioassays, and probabilistic risk modeling in a single framework. This convergence allows the class-specific prioritization of compounds of concern and strengthens the scientific basis for decision-making in sanitation policies. Our findings support recent WHO and UNEP recommendations for the inclusion of AMR monitoring in wastewater surveillance programs. Based on our results, we recommend the implementation of tertiary treatment technologies such as ozonation, powdered activated carbon, or constructed wetlands designed specifically to remove antibiotics. Regulatory agencies should consider establishing effluent discharge thresholds based not only on ecotoxicity but also on minimum inhibitory concentrations (MICs) relevant to resistance selection.

Despite the strengths of this study, some limitations must be acknowledged. This study relied on culture-dependent methods, which may have underestimated ARB diversity and excluded viable, but nonculturable organisms. Additionally, the lack of molecular confirmation of resistance genes limits mechanistic insights. Future studies should include metagenomic analyses, mobile genetic element tracking, and quantification of clinically relevant resistance genes (e.g., *bla_KPC_*, *bla_CTX-M_*, and *qnr*) to better capture AMR dynamics in aquatic systems and inform mitigation strategies. In summary, our data demonstrate that treated effluents, although compliant with physicochemical standards, may still harbor compounds and organisms associated with AMR propagation. By integrating chemical, biological, and statistical approaches, this study advances current understanding and offers a scalable tool for wastewater surveillance in tropical urban environments.

## 4. Materials and Methods

### 4.1. Wastewater Treatment Plant (WWTP)

This study was conducted at a major WWTP located in Curitiba, Paraná, Brazil, which receives domestic wastewater, including hospital sewage effluent and pharmaceutical waste. The plant operates with preliminary screening, a grit chamber, six upflow anaerobic sludge blanket (UASB) reactors, and secondary treatment via aerated and sedimentation lagoons. During the monitoring period, the WWTP had a nominal capacity of 420 L s^−1^. Although this study was conducted in a single WWTP, it is one of the largest and most representative plants in the region, serving approximately 290,000 inhabitants. Thus, these site-specific findings may provide relevant insights for regional assessments of antimicrobial contamination in urban wastewater systems.

### 4.2. Sampling Campaign and Preparation

Effluent sampling was conducted monthly at 10:00 a.m., using a simple sample from November 2022 to March 2023. Effluent was collected after the second lagoon. On each sampling date, four 50 mL sterile samples were collected for chromatographic analysis, and 1000 mL samples were collected in sterile containers for microbiological assessment. All the samples were immediately cooled (4 °C) and transported to the laboratory. For chemical analyses, samples were filtered through 0.45 µm glass fiber membranes (Millipore, Burlington, MA, USA) and stored at 4 °C in the dark until analysis.

### 4.3. Physicochemical Evaluations

The effluent temperature, pH, conductivity, and total soluble solids (TSS) were measured in situ using a portable multiparameter probe (Atra, São Paulo, Brazil). Dissolved oxygen (DO) was measured using a BLE-9100 galvanic probe (Yieryi, Shenzhen, China). Apparent and true colors were measured using an HI727 CheckerHC (Hanna Instruments, Woonsocket, RI, USA). True color measurements were performed after filtering the samples through 0.22 µm membranes.

### 4.4. Antimicrobial Detection

#### 4.4.1. Compound Selection

Thirteen antimicrobials were selected: ciprofloxacin, enrofloxacin, norfloxacin, levofloxacin (fluoroquinolones), azithromycin (macrolide), amoxicillin (β-lactam), meropenem (carbapenem), sulfamethoxazole, sulfadiazine (sulfonamides), tetracycline, oxytetracycline (tetracyclines), and gentamicin (aminoglycosides). The selection was based on multiple criteria: (i) high regional prescription and consumption rates, (ii) frequent detection in Brazilian aquatic environments, (iii) inclusion in the WHO Priority Pathogen List, and (iv) clinical importance in human and veterinary medicine [18,27,28,29,30,31]. This combination ensures the ecological, clinical, and regulatory relevance of the monitored compounds.

#### 4.4.2. Chromatographic Analyses

Sample preparation followed the QuEChERS method [32], using chemicals obtained from Sigma-Aldrich. In this method, 25 mL of the filtered sample was mixed with 5 mL of acetonitrile in sterile 50 mL centrifuge tubes. For quality control, filtered samples were spiked with known quantities of internal standards, ciprofloxacin-d8 hydrochloride hydrate (CIP-d8; final concentration 10 mg L^−1^) and sulfamethoxazole-d4 (SMX-d4; final concentration 10 mg L^−1^), also obtained from Sigma-Aldrich (São Paulo, Brazil), and then extracted using the QuEChERS method. Acetate buffer (6 g MgSO_4_, 1.5 g Na acetate) was added to the mixture, and the tubes were manually shaken and centrifuged (5 min at 3700 rpm). The samples were cleaned by adding 10 mL of supernatant into a 15 mL centrifuge tube with a primary secondary amine (PSA) sorbent and 10 mg MgSO_4_. The tubes were shaken and centrifuged (3700 rpm for 3 min) before the extracts were filtered (0.22 µm) and aliquoted into screw cap vials. A multicompound stock standard solution (1000 µg L^−1^) was added to the samples to estimate recovery rates. A volume of the standard solution was added to the samples to achieve concentrations of 50, 100, and 500 µg L^−1^. Finally, 1 mL aliquots were transferred into screw-cap vials. All extractions were performed at 20 °C.

LC-MS/MS was used to measure the antimicrobial compounds, employing a Xevo TQD triple quadrupole mass spectrometer (Waters Corporation, Milford, MA, USA) with an ESI ionization source and an HPLC Varian SYSLC-240-E (Varian, Santa Clara, CA, USA) with an autosampler. The protocol for drug analysis has been described by Marques et al. [1]. Positive mode analyses were conducted using a ZORBAX SB-C18 column (2.1 mm × 100 mm, 1.8 μm particle size, Agilent Technologies, Santa Clara, CA, USA). A gradient elution process using water (phase A) and acetonitrile (phase B) was used. Calibration curves were constructed using analytical grade drugs. Standard stock solutions (1000 μg mL^−1^) of these compounds were prepared using different compositions of methanol, water, acetonitrile, formic acid, and ammonium formate depending on their solubility. Ten-point calibration curves ranging from 1 to 20 µg L^−1^ were used for calibration, and external standardization was employed to evaluate the linearity of the responses using a linear regression model. Method validation included the evaluation of linearity (R^2^ ≥ 0.95), intraday precision (expressed as RSD < 15%), limits of detection (Table S1), matrix effects, and recovery rates, which ranged from 74% to 89% for the studied compounds. The matrix effect was assessed by comparing the angular coefficients of the calibration curves prepared in the solvent to those of the matrix. The quality parameters for each compound, including retention time, transitions, collision energy, and validation metrics, are summarized in Table S4.

### 4.5. Ecotoxicological Assay

The green alga *Desmodesmus subspicatus* was used to evaluate the effluent toxicity. Cultures were grown in CHU10 medium [33] at 24 ± 2 °C under a 10/14 h light/dark cycle. Assays were performed in 96-well plates with effluent dilutions (0, 6.25, 12.5, 25, 50, and 100%) for 72 h [34]. Algal density was assessed by measuring the absorbance at 750 nm and correlated with the cell count [35]. Each treatment was replicated four times.

### 4.6. Antimicrobial-Resistant Bacteria

#### 4.6.1. Isolation and Screening

Resistant bacteria were assessed using a modified version of the APHA protocols [36], focusing on clinically important resistant organisms. Sampling targeted both community- and hospital-derived bacteria of clinical concern in Brazil, including Enterobacterales resistant to cephalosporins, carbapenems, and non-fermenters, such as *Pseudomonas* and *Acinetobacter* spp. These taxa are among the most frequently detected in Brazilian effluents and appear on the WHO Priority Pathogen List. Samples (1000 mL) were collected and transported in sterile DNA-free containers, filtered through 0.45 µm membranes, and enriched in tryptic soy broth with ceftriaxone or meropenem (1 mg L^−^^1^). After 12 h of incubation (35 °C), the cultures were plated on HiCrome UTI Agar containing the same antibiotics. The plates were incubated for 18–24 h at 35 °C.

#### 4.6.2. Identification and Susceptibility Testing

Isolates were identified using matrix-assisted laser desorption ionization time-of-flight mass spectrometry (MALDI-TOF) with a Microflex MALDI-TOF Biotyper system (Bruker Daltonics Inc., Billerica, MA, USA) according to the manufacturer’s instructions. Antimicrobial susceptibility was determined using disk diffusion (EUCAST guidelines). The tested antimicrobials varied by bacterial species: Enterobacterales were tested for gentamicin, amikacin, ertapenem, meropenem, ceftriaxone, cefepime, sulfamethoxazole-trimethoprim and ciprofloxacin; *Pseudomonas* spp., *Acinetobacter* spp., and *Aeromonas* spp. were tested for meropenem, imipenem, amikacin, gentamicin, cefepime, ciprofloxacin, and ceftazidime; all Gram-negative bacilli underwent polymyxin susceptibility testing using the broth microdilution technique standardized by EUCAST. Phenotypic tests for the detection of extended-spectrum β-lactamases and carbapenemases were performed using the double-disk diffusion test according to Jarlier et al. [37] and the NG Carba immunochromatographic test (NG-Test^®^ Carba-5 (NG Biotech Laboratoires, Guipry, Brittany, France). Quality control was maintained using *Escherichia coli* ATCC 25922 and *Pseudomonas aeruginosa* ATCC 27853. Strains resistant to one or more antimicrobial classes were preserved in TSB with 15% glycerol at −80 °C for further study. As these are culture-based methods, they may underestimate the true diversity of resistant bacteria by excluding viable but nonculturable organisms or resistance genes that are not phenotypically expressed under laboratory conditions.

### 4.7. Statistical Evaluations

Normality and homogeneity of variances were assessed using the Shapiro–Wilk and Bartlett tests, respectively. Temporal changes in physicochemical and antimicrobial variables were analyzed using repeated-measures ANOVA (JMP v10.0), with Mauchly’s test for sphericity and Greenhouse–Geisser corrections when necessary. Differences between treatments in the algal toxicity bioassay were assessed using one-way ANOVA followed by Tukey’s post hoc test (*p* < 0.05) (Tables S5 and S6).

Bivariate and multivariate statistical approaches were used to explore the relationship between antibiotic concentrations and the occurrence of resistant bacteria in the treated effluents. The monthly average concentrations of the antibiotics were grouped into six pharmacological classes: fluoroquinolones, macrolides, carbapenems, sulfonamides, tetracyclines, and aminoglycosides. For each class, concentration levels were categorized as low (<0.1 µg/L), medium (0.1–1.0 µg/L), and high (>1.0 µg/L). These thresholds were defined based on a combination of the observed concentration ranges in the effluent dataset and the environmentally relevant thresholds reported in the literature. The number of clinically relevant resistant bacterial taxa was calculated monthly for each class of antibiotics. Pearson’s correlation analyses were conducted to evaluate the association between the average concentration per class and frequency of resistant bacteria. No statistically significant correlations were observed (*p* > 0.05). Correspondence analysis (CA) was conducted using contingency tables built from resistance occurrences by concentration level. CA ordination showed that macrolides and carbapenems were more frequently associated with high concentrations, whereas fluoroquinolones were aligned at medium levels. Classes such as sulfonamides, tetracyclines, and aminoglycosides showed no consistent associations across concentration levels. The first two dimensions of CA explained 100% of the data variance (Dimension 1:95.7%; Dimension 2:4.3%). All analyses were conducted in R (v4.3.0) using FactoMineR, factoextra, tidyverse, and ggplot2 packages.

### 4.8. Risk Assessment for Ecotoxicity and Antimicrobial Resistance Selection

Risk assessment was performed following USEPA protocols (2001, 2020), considering both ecotoxicological impacts and the risk of resistance selection. The predicted no-effect concentration for ecotoxicity (PNEC_ecotox_) was derived from chronic or acute NOEC, EC50, or LC50 values divided by an assessment factor (AF) of 1000. The ecotoxicological risk was calculated using the hazard quotient (HQ_ecotox_), as follows:

(1) $${PNEC}_{ecotox}=\frac{MEC}{{PNEC}_{ecotox}}$$

where MEC is the measured environmental concentration, and PNEC_ecotox_ is the predicted no-effect concentration for aquatic organisms. To assess mixture toxicity, the analyzed compounds were initially categorized according to their toxic mode of action (TMoA). When the variation in LD_50_ values within a given TMoA group exceeded 10%, the compounds were reassigned to distinct categories to ensure mechanistic consistency [38]. The multisubstance potentially affected fraction (msPAF) for each TMoA group was calculated using a log-normal distribution model, as follows:

(2) $$msPAFTMoA=NORM.DIST\;({log(RISK}_{TMoA}),\;0,\;\sigma_{TMoA},1)$$

where RISK_TMoA_ denotes the cumulative exposure risk of all compounds within the group and σ_TMoA_ is the standard deviation of the group’s log-transformed toxicity values. To derive the total toxicity of the mixture (msPAFTotal), it was assumed that the biological responses of the different TMoA groups were additive. Thus, the integrated effect was calculated as follows:

(3) $$msPAFTotal=1-\prod_{i=1}^{n}\left(1-msPAFTMoA,i\right)$$

Subsequently, the contribution of each chemical group to the overall toxicity of the mixture was determined and expressed as a proportion of total msPAF. Both the single-compound PAF values and msPAFTotal reflect the estimated fraction of species within an ecosystem that can be adversely affected by exposure to individual substances or complex mixtures. A conservative (worst-case) scenario was adopted by considering the highest concentrations and frequencies of occurrence of each compound detected across all the studies included in the assessment (Table S2).

The risk of selecting antimicrobial-resistant bacteria was assessed using the hazard quotient for resistance selection (HQ_res. sel._), calculated as the lowest minimum inhibitory concentration (MIC) value for environmental bacteria divided by an AF of 1000 [24]:

(4) $${HQ}_{res.\;\;sel}=\frac{MEC}{{HQ}_{res,sel}}$$

A probabilistic model adapted from Steen et al. [39] was applied to estimate the integrated risks of the antibiotic mixtures. A log-normal distribution was fitted to the MEC/PNEC_res. sel._ ratios for each antibiotic using five monthly concentration values. Individual probability of exceeding PNEC_res. sel_. (Φ_i_) was calculated, and the cumulative risk of the mixture was estimated using the following expression:

(5) $$\Phi_{total}=1-\prod \left(1-\Phi_{i}\right),\;from\;i-1\;to\;n$$

where n is the number of antibiotics analyzed. This approach allows the quantification of the probability that at least one antibiotic in a mixture poses a risk of resistance selection.

## 5. Conclusions

This study demonstrated that treated domestic effluents, although compliant with the current physicochemical standards, can still contain residual concentrations of antimicrobials and ARB. The consistent detection of multiple antibiotics over five months, along with the isolation of clinically relevant resistant strains, including ESBL and carbapenemase producers, highlights the limitations of conventional wastewater treatment in fully removing micropollutants and resistance determinants. Rather than portraying WWTPs as sources of AMR, our findings emphasize their pivotal roles as control points and environmental sentinels within the One Health Framework. These systems serve as critical surveillance nodes for tracking the dissemination of resistance in urban aquatic environments, and must be integrated into AMR monitoring and policy initiatives. WWTPs receive contaminants from human activities and play a key role in reducing their release into natural bodies of water. Monitoring treated effluents provides critical information for public health and environmental risk assessments, especially from the One Health Perspective.

This study advances our understanding of AMR dissemination in urban aquatic environments by integrating chemical, microbiological, toxicological, and probabilistic approaches. Notably, the analysis of mixture-level toxicity and resistance selection revealed substantial cumulative risks that would be overlooked by single-compound assessments, underscoring the need to prioritize mixture-based approaches in environmental regulation. This highlights the need for the adoption of complementary advanced treatment technologies, such as ozonation, activated carbon filtration, or nature-based solutions, such as constructed wetlands, to enhance the removal of antimicrobial residues and reduce resistance selection pressures. Finally, we encourage the inclusion of AMR-related endpoints in environmental monitoring frameworks and recommend that regulatory thresholds consider not only ecotoxicological but also microbiological criteria such as MICs. Strengthening wastewater surveillance is essential to protect aquatic biodiversity and public health in rapidly urbanizing tropical regions.

## Figures and Tables

**Table 1:** Maximum (Max), minimum (Min) and median (Me) concentrations of antimicrobials found in effluents from a WWTP in Curitiba, Brazil from November 2022 to March 2023. The typical use category for each compound (H = human, V = veterinary, and H + V = both) is indicated.

Concentration (ng L^−1^)	CIP	ENR	NOR	LEV	AZI	AMX	MEP	SMX	SDZ	TC	OTC	GEN
Min	71.2	21.5	42.1	94.2	100	145	199	138	9.85	78.6	275	221
Max	303	125	149	286	211	402	612	426	85.2	285	745	432
Me	99.9	59.4	78.6	192	160	258	375	281	60.9	149	360	381
Use Category	H + V	V	H + V	H + V	H + V	H + V	H + V	H + V	H + V	H + V	H + V	H + V
Antibiotic class	Fluoroquinolone				Macrolide	β-lactam	Carbapenem	Sulfonamide		Tetracycline		Aminoglycoside

CIP, ciprofloxacin; ENR, enrofloxacin; NOR, norfloxacin; LEV, levofloxacin; AZI, azithromycin; AMX, amoxicillin; MEP, meropenem; SMX, sulfamethoxazole; SDZ, sulfadiazine; TC, tetracycline; OTC, oxytetracycline; GEN, gentamicin.

**Table 2:** Toxicological contribution of antibiotics to cumulative mixture risk (msPAF_Total_) in acute and chronic exposure scenarios based on mode of action (μTMoA) and RISKTMoA calculations.

Acute											
	SMX	SDZ	CIP	ENR	NOR	LEV	OTC	TC	AZI	AMX	MER
μTMoA	6.15	6.92	2.37	6.60	7.23	5.73	6.45	6.13	4.96	6.91	7.41
RISKTMoA	45.80	7.71	54.80	9.88	11.89	30.58	64.41	25.10	29.43	37.74	48.40
msPAF	1.00	1.00	0.55	1.00	1.00	0.99	1.00	1.00	0.99	0.99	0.96
Relative contribution	0.28	3.34 × 10^−7^	92.37	0.00	0.66	1.40	0.05	0.02	2.10	1.88	7.75
msPAFTotal		0.50									
**Chronic**											
	**SMX**	**SDZ**	**CIP**	**ENR**	**NOR**	**LEV**	**OTC**	**TC**	**AZI**	**AMX**	**MER**
μTMoA	5.15	5.91	1.51	5.60	6.23	4.73	5.45	5.13	3.96	5.82	6.41
RISKTMoA	54.69	9.01	86.17	11.64	13.80	37.05	76.24	29.99	36.87	44.81	55.95
msPAF	0.96	1.00	0.55	0.99	0.99	0.96	0.99	1.00	0.93	0.97	0.92
Relative contribution	2.49	2.82	78.19	0.00	2.35	6.84	0.82	0.41	11.93	4.94	13.77
msPAFTotal		0.57									

**Table 3:** Probabilistic risk estimates (Φi) of antimicrobial resistance selection for each antibiotic detected in WWTP effluents from Curitiba, Brazil, based on log-normal distributions of MEC/PNEC_res. sel._.

	SMX	SDZ	CIP	ENR	NOR	LEV	OTC	TC	AZI	AMX	MER
PNEC_res. sel._	16	16	0.064	0.064	0.5	0.25	0.5	1	0.25	0.25	0.064
HQ_res. sel._	3.33	17.61	2033.13	1019.06	171.94	700.56	830.5	153.72	583.36	1043.44	5601.88
Φ_i_	1	1	1	1	1	1	1	1	1	1	1
Combined Risk (Φ_total_)		1									

Φ_i_ Probability of exceeding PNEC_res. sel._

**Table 4:** Antimicrobial-resistant bacteria detected in treated effluent samples.

Group		Bacterial Specie	Sampling Months
Enterobacterales carbapenem-resistant	Carbapenemase detected *	*Enterobacter cloacae* complex	Dec and Feb
		*Escherichia coli*	Feb and Mar
		*Klebsiella pneumoniae* complex	Nov to Mar
		*Raoultella* sp.	Dec
	No carbapenemase detected *	*Enterobacter cloacae* complex	Nov
		*Klebsiella pneumoniae* complex	Feb
Enterobacterales third-generation cephalosporin-resistant	ESBL detected **	*Citrobacter freundii* complex	Nov and Dec
		*Enterobacter cloacae* complex	Nov and Feb
		*Escherichia coli*	Nov to Mar
		*Klebsiella oxytoca* complex	Dec
		*Klebsiella pneumoniae* complex	Nov to Mar
		*Providencia alcalifaciens*	Dec

* Phenotypic immunochromatographic test. ** Using phenotypic double-disk diffusion test. Nov = November, Dec = December, Feb = February, Mar = March.

## Data Availability

All data supporting the findings of this study are available from the corresponding author upon reasonable request.

## Supplementary Materials

The following supporting information can be downloaded at: https://www.mdpi.com/article/10.3390/antibiotics14080836/s1, Figure S1: Physicochemical characteristics of effluents from a WWTP in Curitiba, Brazil, from November 2022 to March 2023. Values represent the mean ± standard deviation (n = 4). Different lowercase letters indicate statistically significant differences between months (*p* < 0.05, Tukey’s HSD test); Table S1: Growth (number of cells × 10^5^) of *Desmodesmus subspicatus* exposed to serial dilutions of effluents from a WWTP in Curitiba, Brazil, from November 2022 to March 2023; Table S2: Acute and chronic msPAF and relative contributions of antibiotics to mixture toxicity; Table S3: Occurrence of antibiotic-resistant bacteria in treated effluent; Table S4: Linearity parameters for validation of the LC-MS/MS method; Table S5: Repeated-measures ANOVA for the effects of time (months) on physicochemical parameters of WWTP effluents; Table S6: Repeated-measures ANOVA for the effects of time (months) on antimicrobial concentrations in WWTP effluents.
