# Supplementary material for: Occurrence and Risk Assessment of Antimicrobials and Resistant Bacteria in Treated Sewage Effluents in South Brazil
Source: Antibiotics (Basel). 2025 Aug 18;14(8):836. doi: 10.3390/antibiotics14080836 (PMC12383130; doi:10.3390/antibiotics14080836)
Supplement: Supplementary file 1 [file antibiotics-14-00836-s001.zip › antibiotics-3778903-supplementary.pdf]

Article

Occurrence and Risk Assessment of Antimicrobials and Resistant Bacteria in Treated Sewage Effluents in South Brazil

Keite da Silva Nogueira [1,2], Ana Paula Tomaz de Oliveira[2], Gabrielly Cristina Kubis [3], Raizza Zorman Marques [3], Nicole Geraldine de Paula Marques Witt [3], Bárbara Zanicotti Leite [4], Marcelo Pedrosa Gomes [3,*]

[1] Departamento de Patologia Básica, Setor de Ciências Biológicas, Universidade Federal do Paraná, Avenida Coronel Francisco H. dos Santos, 100, Centro Politécnico Jardim das Américas, CEP 19031, 81531-980, Curitiba, Paraná, Brazil.
[2] Laboratório de Bacteriologia, Complexo Hospital de Clínicas, Universidade Federal do Paraná, Rua Padre Camargo, 280, CEP 80060-240, Curitiba, Paraná, Brazil.
[3] Laboratório de Fisiologia de Plantas sob Estresse, Departamento de Botânica, Setor de Ciências Biológicas, Universidade Federal do Paraná, Avenida Coronel Francisco H. dos Santos, 100, Centro Politécnico Jardim das Américas, C.P. 19031, 81531-980, Curitiba, Paraná, Brazil.
[4] Companhia de Saneamento do Paraná (SANEPAR) - Sede Administrativa, Rua Engenheiros Rebouças, 1376, Rebouças, CEP 80215-900, Curitiba, Paraná, Brazil

* Correspondence: marcelo.gomes@ufpr.br

1. Physicochemical characteristics

The effluent pH ranged from approximately 6.3 to 7.2 (Fig. 1A). Conductivity increased from 757 µS cm^{-1} in November 2022 to 813 µS cm^{-1} in February 2023, reaching its peak at 981 µS cm^{-1} in March 2023 (Fig. 1B). The highest effluent temperature was recorded in January 2023 at 29.7°C (Fig. 1C). Similarly, total soluble solids (TSS) reached their maximum levels in March 2023 (Fig. 1D). The apparent color of the effluent ranged from 235 to 500 PCU, peaking in February 2023 (Fig. 1E), whereas the real color varied from 26 to 79 PCU, with the highest value in March 2023 (Fig. 1E). The concentration of dissolved oxygen (DO) was highest in November 2022, ranging from 0.57 to 9.76 mg L^{-1} (Fig. 1F). Chemical oxygen demand (COD) and biochemical oxygen demand (BOD) showed no significant changes over time, ranging from 148.5 to 185.5 mg O_2 L^{-1} and 54.0 to 67.5 mg O_2 L^{-1}, respectively (Fig. 1G and H).

The physicochemical parameters of the WWTP effluent, including pH, conductivity, temperature, total soluble solids (TSS), color, and dissolved oxygen (DO), varied throughout the study period within the limits set by the Brazilian regulations on effluent standards (Resolução N. 430, de 13 de Maio de 2011, 2011). The pH values ranged from 6.3 to 7.2, which was within the acceptable limits for discharge into aquatic environments, suggesting that the effluent was relatively neutral. However, the increase in conductivity and TSS, particularly the peak values in March 2023 (Fig. 1S), indicates an increase

in dissolved and suspended solids, which could affect aquatic life by altering the water quality and light penetration.

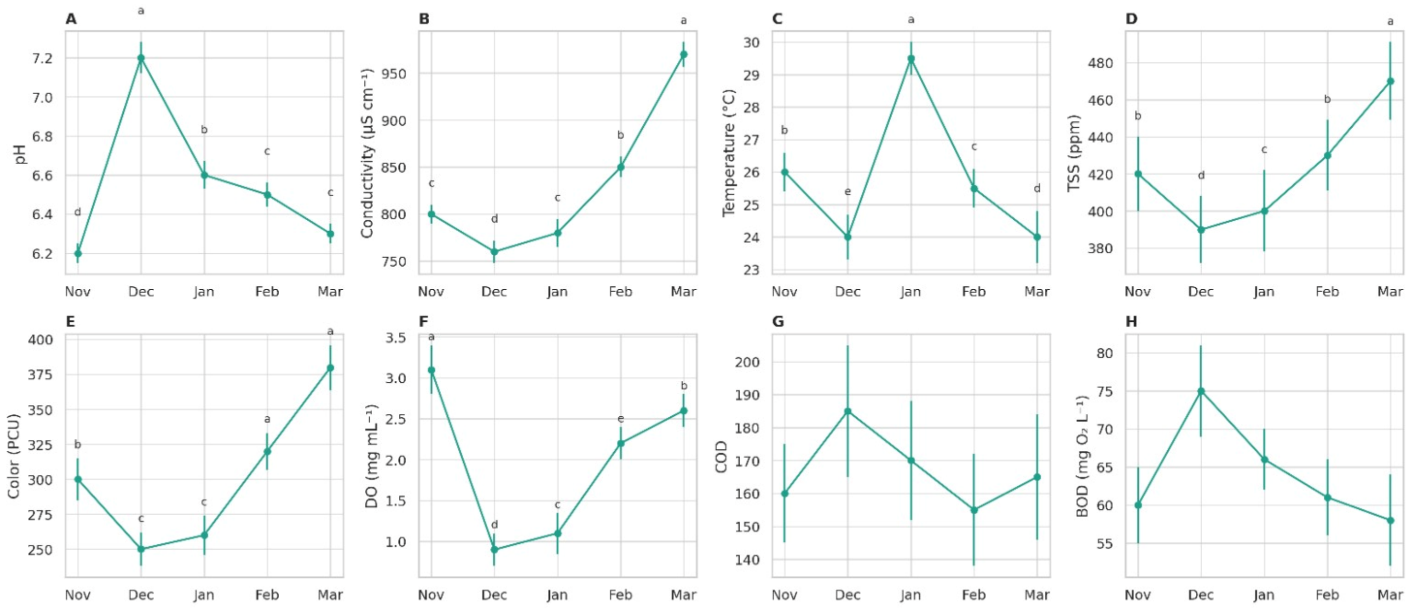

Figure 1S. Physicochemical characteristics of effluents from a WWTP in Curitiba, Brazil, from November 2022 to March 2023. Values represent the mean ± standard deviation (n = 4). Different lowercase letters indicate statistically significant differences between months ($p < 0.05$, Tukey's HSD test).

Table S1. Growth (number of cells × 105) of Desmodesmus subspicatus exposed to serial dilutions of effluents from a WWTP in Curitiba, Brazil from November 2022 to March 2023.

**Treatment**	**Dilution**	**Nov/2022**		**Dec/2022**		**Jan/2023**		**Feb/2023**		**Mar/2023**	
		Mean	SD	Mean	SD	Mean	SD	Mean	SD	Mean	SD
Control	-	8.57^{a}	0.29	8.57^{a}	0.29	8.40^{a}	0.50	8.57^{a}	0.29	8.40^{a}	0.50
Effluent	6.25%	8.73^{a}	0.29	8.57^{a}	0.29	8.40^{a}	0.50	8.57^{a}	0.29	8.40^{a}	0.50
Effluent	12.5%	8.57^{a}	0.29	8.57^{a}	0.58	8.57^{a}	0.29	8.57^{a}	0.29	8.57^{a}	0.29
Effluent	25%	8.73^{a}	0.58	8.57^{a}	0.29	8.40^{a}	0.00	8.73^{a}	0.58	8.40^{a}	0.50
Effluent	50%	8.57^{a}	0.29	8.40^{a}	0.50	8.57^{a}	0.29	8.57^{a}	0.29	8.40^{a}	0.50
Effluent	100%	7.57^{b}	0.29	6.23^{b}	0.58	6.23^{b}	0.58	7.57^{b}	0.29	6.23^{b}	0.58
F ratio		4.02		11.61		13.73		3.90		9.72	
P		<0.05		<0.001		<0.001		<0.05		<0.001	

Values followed by the same letter in the columns do not differ significantly according to Tukey's test ($P < 0.05$) ($n = 4$).

Table S2. Acute and Chronic msPAF and Relative Contributions of Antibiotics to Mixture Toxicity.

Acute																				
**Chemical**	**Reference**	**NOEC mg/L**	**NOEC ng/L (acute)**	**Test Species**	**End point**	**Test duration**	**PNEC ng/L**	**Concentration ng/L**	**HQ eco-tox**	**EC5**	**LOGEC5**	**μ_{TMoA}**	**σ_{TMoA}**	**RISK TMoA**	**Group**	**PAF**	**MS PAF**	**Relative Contribution**	**Product**	**msPAF TOTAL**
SMX	ECOSAR	6.62	6620000	Green algae	EC50	96h	6620	281.81	0.04	662000	5.820858	6.153503	1.234446	45.79668	1	0.001349152	0.9986508	0.27810092	0.514887	0.48513
	ECOSAR	1.87	1870000	Daphnids	LC50	48h	1870	281.81	0.15	187000	5.271842									
	ECOSAR	410.76	410760000	Fish	LC50	96h	410760	281.81	0.00	41076000	7.613588									
	10.1016/j.chemosphere.2020.127407	12.56	12560000	*Lemna minor*	IC50	7 dias	12560	281.81	0.02	1256000	6.09899									
	10.1016/j.chemosphere.2020.127407	43.97	43970000	*Daphnia magna*	EC50	48h	43970	281.81	0.01	4397000	6.643156									
	10.1016/j.envint.2006.11.017	189.2	189200000	*Daphnia magna*	EC50	48h	189200	281.81	0.00	18920000	7.276921									
	10.1016/j.chemosphere.2010.04.011	0.05	50000	*Aliivibrio fischeri*	EC50	30 min	50	281.81	5.64	5000	3.698997									
	10.1016/j.jhazmat.2016.02.013	100	100000000	*Aliivibrio fischeri*	EC50	30 min	100000	281.81	0.00	10000000	7									
	10.1007/s11356-024-34659-y	693.9	693900000	*Aliivibrio fischeri*	EC50	30 min	693900	281.81	0.00	69390000	7.841297									
	10.1007/s11356-024-34659-y	17.1	17100000	*Escherichia coli*	EC50	24h	17100	281.81	0.02	1710000	6.232996									
	10.1007/s11356-024-34659-y	2.11	2110000	*Lemna minor*	EC50	7d	2110	281.81	0.13	211000	5.324282									
	10.1007/s11356-024-34659-y	49.4	49400000	*Daphnia magna*	EC5	48h	49400	281.81	0.01	4940000	6.693727									
	10.1016/j.aquatox.2014.08.015	0.00656	6560	*Microcystis aeruginosa*	NOAEC	72h	6.56	281.81	42.96	6560	3.816904									
	https://link.springer.com/article/10.1007/s10646-008-0209-x	123.1	123100000	*Daphnia magna*	EC50	48h	123100	281.81	0.00	12310000	7.090258									
	https://link.springer.com/article/10.1007/s10646-008-0209-x	70.4	70400000	*Moina macrocopa*	EC50	48h	70400	281.81	0.00	7040000	6.847573									
	https://doi.org/10.1016/j.chemosphere.2004.07.017	1.53	1530000	*Selenastrum capricornutum*	EC50	72h	1530	281.81	0.18	153000	5.184691									

	ECOSAR	10.25	102500000	Green algae	EC50	96h	10250	53.31	0.01	1025000	6.010724	6.916457	0.877334	7.707703	2	1.65684E-09	0.999999998	3.41525E-07
	ECOSAR	1.88	1880000	Daphnids	LC50	48h	1880	53.31	0.03	188000	5.274158							
	ECOSAR	1516.1	1516100000	Fish	LC50	96h	1516100	53.31	0.00	151610000	8.180728							
	https://doi.org/10.1016/j.chemosphere.2009.02.002	212	212000000	*Daphnia magna*	EC50	48h	212000	53.31	0.00	21200000	7.326336							
**SDZ**	https://doi.org/10.1016/S0045-6535(99)00443-9	221	221000000	*Daphnia magna*	EC50	48h	221000	53.31	0.00	22100000	7.344392							
	https://doi.org/10.1016/j.chemosphere.2013.01.029	27.2	27200000	*Daphnia magna*	EC50	48h	27200	53.31	0.00	2720000	6.434569							
	https://doi.org/10.1016/j.chemosphere.2013.01.029	188	188000000	*Daphnia magna*	EC50	48h	188000	53.31	0.00	18800000	7.274158							
	https://doi.org/10.1016/j.chemosphere.2013.01.029	310	310000000	*Daphnia magna*	EC50	48h	310000	53.31	0.00	31000000	7.491362							
	https://doi.org/10.1007/s00128-017-2153-z	81.6	81600000	*Scenedesmus obliquus*	EC50	96h	81600	53.31	0.00	8160000	6.911169							
	ECOSAR	162.16	162160000	Green algae	EC50	96h	162160	130.12	0.00	16216	4.209944	2.374727	1.996188	54.79366	3	0.448109093	0.551890907	92.36877989
	ECOSAR	124.04	124040000	Daphnids	LC50	48h	124040	130.12	0.00	12404	4.093562							
	ECOSAR	1313.14	1313140000	Fish	LC50	96h	1313140	130.12	0.00	131314	5.118311							
	10.1016/j.aquatox.2014.08.015	0.0153	1530	*Microcystis aeruginosa*	EC10	72h	1.53	130.12	85.05	0.765	-0.116344							
**CIP**	10.1016/j.aquatox.2014.08.015	0.1626	162600	*Microcystis aeruginosa*	EC50	72h	162.6	130.12	0.80	16.26	1.211121							
	https://doi.org/10.1016/j.aquatox.2014.08.015	241	241000000	*Pseudomonas putida*	EC50	16min	241000	130.12	0.00	24100	4.382017							
	10.1016/j.chemosphere.2014.06.062	23	23000000	*Chlorella* sp	EC50	72h	23000	130.12	0.01	2300	3.361728							
	10.1016/j.chemosphere.2014.06.062	71	71000000	*Moina macrocopa*	EC50	48h	71000	130.12	0.00	7100	3.851258							
	https://doi.org/10.1590/s2175-97902019000217661	0.1724	17240	*Microcystis aeruginosa*	EC50	48h	17.24	130.12	7.55	1.724	0.236537							
	https://doi.org/10.1590/s2175-97902019000217661	0.1356	13560	*Microcystis*	EC50	48h	13.56	130.12	9.60	1.356	0.13226							

				panniformis														
	https://doi.org/10.1002/etc.678	0.0102	10200	*Anabaena flos-aquae*	EC50	72h	10.2	130.12	12.76	1.02	0.0086							
	https://doi.org/10.3390/jox14020042	0.01356	13560	*Microcystis panniformis*	EC50	96h	13.56	130.12	9.60	1.356	0.13226							
	https://doi.org/10.1093/jac/46.suppl_1.53	0.005	5000	*Microcystis aeruginosa*	EC50	72h	5	130.12	26.02	0.5	-0.30103							
	https://doi.org/10.1016/j.ecoenv.2014.11.021	11.3	11300000	*Raphidocelis subcapitata*	EC50	72h	11300	130.12	0.01	1130	3.053078							
	https://doi.org/10.1016/j.chemosphere.2014.02.003	4.45	4450000	*Daphnia curvirostris*	EC50	48h	4450	130.12	0.03	445	2.64836							
	10.1016/j.chemosphere.2020.127823	36	36000000	*Ceriodaphnia dubia*	EC50	48h	36000	130.12	0.00	3600	3.556303							
	https://doi.org/10.1016/j.envpol.2021.116779	620	620000000	*Danio rerio*	EC50	66h	620000	130.12	0.00	62000	4.792392							
	ECOSAR	561	561000000	Green algae	EC50	96h	561000	65.22	0.00	56100000	7.748963	6.602461	1.111821	9.878134	4	8.29204E-06	0.9999917	1.71E-03
	ECOSAR	505	505000000	Daphnids	LC50	48h	505000	65.22	0.00	50500000	7.703291							
	ECOSAR	4900	4900000000	Fish	LC50	96h	4900000	65.22	0.00	490000000	8.690196							
	10.1016/j.chemosphere.2014.06.062	111	111000000	*Chlorella* sp	EC50	72h	111000	65.22	0.00	11100000	7.045323							
	10.1016/j.chemosphere.2014.06.062	69	69000000	*Moina macrocopa*	EC50	48h	69000	65.22	0.00	6900000	6.838849							
**ENR**	https://link.springer.com/article/10.1007/s10646-008-0209-x	56.7	56700000	*Daphnia magna*	EC50	48h	56700	65.22	0.00	5670000	6.753583							
	https://doi.org/10.1002/etc.678	0.173	173000	*Anabaena flos-aquae*	EC50	72h	173	65.22	0.38	17300	4.238046							
	https://doi.org/10.1897/04-210R.1	3.1	3100000	*Raphidocelis subcapitata*	EC50	72h	3100	65.22	0.02	310000	5.491362							
	https://doi.org/10.1002/wer.1631	7.9	7900000	*Daphnia magna*	EC50	48h	7900	65.22	0.01	790000	5.897627							
	https://doi.org/10.1016/j.etap.2019.103295	16.72	16720000	*Daphnia magna*	EC50	48h	16720	65.22	0.00	1672000	6.223236							
	https://doi.org/10.1016/j.chemosphere.2014.02.003	16.34	16340000	*Daphnia magna*	EC50	48h	16340	65.22	0.00	1634000	6.213252							

	https://doi.org/10.1016/j.chemosphere.2014.02.003	4.33	4330000	*Daphnia curvirostris*	EC 50	48h	4330	65.22	0.02	433000	5.636488							
	10.1016/j.chemosphere.2020.127823	60	60000000	*Ceriodaphnia dubia*	EC 50	48h	60000	65.22	0.00	6000000	6.778151							
	https://doi.org/10.1080/02757540.2021.1974007	150	150000000	*Danio rerio*	EC 50	96h	150000	65.22	0.00	15000000	7.176091							
	ECOSAR	43800	43800000000	Green algae	EC 50	96h	43800000	85.97	0.00	4380000000	9.641474	7.230115	1.942209	11.89054	5	0.003198822	0.996801178	0.659373518
	ECOSAR	140000	1.4E+11	Daphinids	LC 50	48h	140000000	85.97	0.00	1.4E+10	10.14613							
	ECOSAR	309000	3.09E+11	Fish	LC 50	96h	309000000	85.97	0.00	3.09E+10	10.48996							
	https://doi.org/10.1038/srep40385	107.6	107600000	*Daphnia magna*	LC 50	96h	107600	85.97	0.00	10760000	7.031812							
	https://doi.org/10.1038/srep40385	175.8	175800000	*Daphnia magna*	LC 50	48h	175800	85.97	0.00	17580000	7.245019							
	10.1007/s10646-009-0334-1	38.49	38490000	*Scenedesmus obliquus*	EC 50	48h	38490	85.97	0.00	3849000	6.585348							
**NOR**	https://doi.org/10.1016/j.chemosphere.2004.07.017	16.6	16600000	*Scenedesmus obliquus*	EC 50	72h	16600	85.97	0.01	1660000	6.220108							
	https://doi.org/10.1016/j.chemosphere.2004.07.017	4.01	4010000	*Scenedesmus obliquus*	NOEC	72h	4010	85.97	0.02	4010000	6.603144							
	https://doi.org/10.1016/j.chemosphere.2004.07.017	10.4	10400000	*Chlorella vugaris*	EC 50	72h	10400	85.97	0.01	1040000	6.017033							
	https://doi.org/10.1016/j.chemosphere.2004.07.017	4.02	4020000	*Chlorella vugaris*	NOEC	72h	4020	85.97	0.02	4020000	6.604226							
	https://doi.org/10.1016/j.watres.2013.01.020	5.6	5600000	*Anabaena CPB 4337*	EC 50	72h	5600	85.97	0.02	560000	5.748188							
	https://doi.org/10.1016/j.aquatox.2021.105826	0.03479	34790	*Microcystis aeruginosa*	EC 50	72h	34.79	85.97	2.47	3479	3.541454							
	https://doi.org/10.1016/j.envpol.2021.116779	1311	1311000000	*Danio rerio*	EC 50	66h	1311000	85.97	0.00	131100000	8.117603							
	10.1016/j.scitotenv.2020.139499	0.1	1000000	*Pseudanabaena sp*	LC 50	**8d**	1000	175.14	0.18	100000	5	5.726603	1.411127	30.58358	6	0.006786031	0.993213969	1.398805266
**LEV**	https://doi.org/10.1080/26395940.2022.2130825	0.4376	437600	*Microcystis aeruginosa*	EC 50	96h	437.6	175.14	0.40	43760	4.641077							
	https://doi.org/10.1016/j.watres.2013.01.020	4.8	4800000	*Anabaena CPB 4337*	EC 50	72h	4800	175.14	0.04	480000	5.681241							

	https://doi.org/10.1897/04-210R.1	7.4	7400000	*Raphidocelis subcapitata*	EC50	72h	7400	175.14	0.02	740000	5.869232							
	https://doi.org/10.1016/j.chemosphere.2020.127823	28	28000000	*Daphnia magna*	EC50	48h	28000	175.14	0.01	2800000	6.447158							
	https://doi.org/10.1002/wer.1631	19.5	19500000	*Daphnia magna*	EC50	48h	19500	175.14	0.01	1950000	6.290035							
	https://doi.org/10.1016/j.ecoenv.2021.112778	15.11	15110000	*Daphnia magna*	EC50	48h	15110	175.14	0.01	1511000	6.179264							
	10.1016/j.chemosphere.2020.127823	35	35000000	*Ceriodaphnia dubia*	EC50	48h	35000	175.14	0.01	3500000	6.544068							
	https://doi.org/10.1897/04-210R.1	0.0079	79000	*Microcystis aeruginosa*	EC50	**5d**	79	175.14	2.22	7900	3.897627							
	https://doi.org/10.1897/04-210R.1	0.051	51000	*Lemna menor*	EC50	7d	51	175.14	3.43	5100	3.70757							
	https://doi.org/10.1016/j.envpol.2021.116779	5437	5437000000	*Danio rerio*	EC50	66h	5437000	175.14	0.00	543700000	8.735359							
	ECOSAR	7.95	7950000	Green algae	EC50	96h	7950	415.25	0.05	795000	5.900367	6.446755	1.088803	64.41225	7	0.000218888	0.999781112	0.045119344
	ECOSAR	4.44	4440000	Daphinids	LC50	48h	4440	415.25	0.09	444000	5.647383							
	ECOSAR	47.67	47670000	Fish	LC50	96h	47670	415.25	0.01	4767000	6.678245							
	https://doi.org/10.1016/S0045-6535(99)00443-9	100	100000000	*Daphnia magna*	LOEC	48h	100000	415.25	0.00	100000000	8							
	https://doi.org/10.1016/j.etap.2013.07.019	127.6	127600000	*Danio rerio*	EC50	72h	127600	415.25	0.00	12760000	7.105851							
**OTC**	https://link.springer.com/article/10.1007/s10646-008-0209-x	621.2	621200000	*Daphnia magna*	EC50	48h	621200	415.25	0.00	62120000	7.793231							
	https://link.springer.com/article/10.1007/s10646-008-0209-x	126.7	126700000	*Moina macrocopa*	EC50	48h	126700	415.25	0.00	12670000	7.102777							
	https://link.springer.com/article/10.1007/s10646-008-0209-x	110.1	110100000	*Oryzias latipe*	LC50	96h	110100	415.25	0.00	11010000	7.041787							
	https://doi.org/10.1016/j.chemosphere.2004.07.017	0.342	342000	*Scenedesmus obliquus*	EC50	72h	342	415.25	1.21	34200	4.534026							
	https://doi.org/10.1016/j.chemosphere.2004.07.017	0.183	183000	*Scenedesmus obliquus*	NOEC	72h	183	415.25	2.27	183000	5.262451							
	https://doi.org/10.1016/j.chemosphere.2004.07.017	7.05	7050000	*Chlorella vugaris*	EC50	72h	7050	415.25	0.06	705000	5.848189							

TC	ECOSAR	3.3	3300000	Green algae	EC50	96h	3300	153.72	0.05	330000	5.518514	6.125078	1.03951	25.09682	8	7.57319E-05	0.999924268	0.01561063
	ECOSAR	2.87	2870000	Daphnids	LC50	48h	2870	153.72	0.05	287000	5.457882							
	ECOSAR	27.09	27090000	Fish	LC50	96h	27090	153.72	0.01	2709000	6.432809							
	https://doi.org/10.1016/S0045-6535(99)00443-9	340	340000000	*Daphnia magna*	NOEC	48h	340000	153.72	0.00	340000000	8.531479							
	https://doi.org/10.1016/j.ecoenv.2019.02.063	7.73	7730000	*Chlorella vulgaris*	EC50	96h	7730	153.72	0.02	773000	5.888179							
	https://doi.org/10.3390/agronomy12102497	10	10000000	*Chlorella pyrenoidosa*	EC50	96h	10000	153.72	0.02	1000000	6							
	https://www.researchgate.net/publication/314278553_Ecotoxicity_of_selected_antibiotics_for_organisms_of_aquatic_and_terrestrial_ecosystems	1.82	1820000	*Pseudokirchneriella subcapitata*	EC50	72h	1820	153.72	0.08	182000	5.260071							
	https://www.researchgate.net/publication/314278553_Ecotoxicity_of_selected_antibiotics_for_organisms_of_aquatic_and_terrestrial_ecosystems	8.16	8160000	*Daphnia magna*	EC50	48h	8160	153.72	0.02	816000	5.91169							
AZI	ECOSAR		21900000	Fish	LC50	96h	21900	145.84	0.01	2190000	6.340444	4.955485	1.204023	29.43001	9	0.010209248	0.989790752	2.104433449
	ECOSAR		3020000	Daphnids	LC50	48h	3020	145.84	0.05	302000	5.480007							
	ECOSAR		1870000	Green algae	EC50	96h	1870	145.84	0.08	187000	5.271842							
	https://doi.org/10.1016/j.emcon.2019.08.004	226.66	226660000	*Vibrio fischeri*	EC50	15min	226660	145.84	0.00	22666000	7.355375							
	10.5281/zenodo.7991973	1.3	1300000	*Daphnia magna*	EC50	48h	1300	145.84	0.11	130000	5.113943							
	https://doi.org/10.1016/j.ecoenv.2021.112553	0.018	18000	*Raphidocelis subcapitata*	EC10	72h	18	145.84	8.10	9000	3.954243							
	https://doi.org/10.1016/j.ecoenv.2021.112553	0.026	26000	*Raphidocelis subcapitata*	EC20	72h	26	145.84	5.61	6500	3.812913							
	https://doi.org/10.1016/j.ecoenv.2021.112553	0.051	51000	*Raphidocelis subcapitata*	EC50	72h	51	145.84	2.86	5100	3.70757							
	https://doi.org/10.1016/j.ecoenv.2021.112553	0.033	33000	*Raphidocelis subcapitata*	LOEC	72h	33	145.84	4.42	33000	4.518514							

	https://doi.org/10.1016/j.ecoenv.2021.112553	0.01	10000	*Raphidocelis subcapitata*	NOEC	72h	10	145.84	14.58	10000	4							
	ECOSAR	14.91	14910000	Green algae	EC50	96h	14910	260.86	0.02	14910000	6.173478	6.911871	1.904674	37.74086	10	0.009131882	0.990868118	1.882355883
	ECOSAR	2.89	2890000	Daphnids	LC50	48h	2890	260.86	0.09	289000	5.460898							
	ECOSAR	37.02	37020000	Fish	LC50	96h	37020	260.86	0.01	3702000	6.568436							
	https://doi.org/10.1016/j.etap.2013.07.019	132.4	132400000	*Danio rerio*	EC50	48h	132400	260.86	0.00	13240000	7.121888							
	https://doi.org/10.1016/j.ecoenv.2020.110207	25	25000000	*Raphidocelis subcapitata*	LOEC	96h	25000	260.86	0.01	25000000	7.39794							
**AMX**	10.5281/zenodo.7991973	10.3	10300000	*Daphnia magna*	EC50	48h	10300	260.86	0.03	1030000	6.012837							
	https://doi.org/10.3390/toxics9080196	27.2	272000000	*Daphnia magna*	NOEC	**21 d**	272000	260.86	0.00	272000000	8.434569							
	https://doi.org/10.3390/toxics9080196	21.8	218000000	*Oryzias latipes*	NOEC	**40 d**	218000	260.86	0.00	218000000	8.338456							
	https://link.springer.com/article/10.1007/s10646-008-0209-x	3597	3597000000	*Vibrio fischeri*	EC50	15min	3597000	260.86	0.00	3597000000	9.55594							
	https://link.springer.com/article/10.1007/s002449900435	0.0037	3700	*Microcystis aeruginosa*	EC50	72h	3.7	260.86	70.50	370	2.568202							
	https://link.springer.com/article/10.1007/s002449900435	250	250000000	*Selenastrum capricornutum*	NOEC	72h	250000	260.86	0.00	250000000	8.39794							
	ECOSAR	14000	14000000000	Green algae	EC50	96h	14000000	358.52	0.00	14000000000	9.146128	7.40746	2.727716	48.39986	11	0.0376097	0.9623903	7.75249198
	ECOSAR	8230	8230000000	Daphnids	LC50	48h	8230000	358.52	0.00	823000000	8.9154							
MER	ECOSAR	101000	1.01E+11	Fish	LC50	96h	101000000	358.52	0.00	1.01E+10	10.00432							
	https://doi.org/10.1016/j.envres.2024.119409	0.033	33000	*Lemna minor*	EC20	7 dias	33	350.80	10.63	8250	3.916454							
	https://doi.org/10.1016/j.envres.2024.119409	1.135	1135000	*Lemna minor*	EC50	7 dias	1135	350.80	0.31	113500	5.054996							
**Cronic**																		

Chemical	Reference	NOEC mg/L	NOEC ng/L (chronic)	Test Species	End point	Test duration	PNEC ng/L	Concentration ng/L	HQ eco-tox	EC5	LOGEC5	μT MoA	σT MoA	RISK TMoA	Group	PAF	MSPAF	Relative Contribution	Produto	msPAF TOTAL
	ECOSAR	6.62	662000	Green algae	EC50	96h	662	281.81	0.43	66200	4.820858	5.153503	1.234446	54.68319	1	0.014258907	0.985741093	2.488119791	0.42692	0.57308
	ECOSAR	1.87	187000	Daphinids	LC50	48h	187	281.81	1.51	18700	4.271842									
	ECOSAR	410.76	41076000	Fish	LC50	96h	41076	281.81	0.01	4107600	6.613588									
	10.1016/j.chemosphere.2020.127407	12.56	1256000	*Lemna minor*	IC50	7 dias	1256	281.81	0.22	125600	5.09899									
	10.1016/j.chemosphere.2020.127407	43.97	4397000	*Daphnia magna*	EC50	48h	4397	281.81	0.06	439700	5.643156									
	10.1016/j.envint.2006.11.017	189.2	18920000	*Daphnia magna*	EC50	48h	18920	281.81	0.01	1892000	6.276921									
	10.1016/j.chemosphere.2010.04.011	0.05	5000	*Aliivibrio fischeri*	EC50	30 min	5	281.81	56.36	500	2.69897									
	10.1016/j.jhazmat.2016.02.013	100	10000000	*Aliivibrio fischeri*	EC50	30 min	10000	281.81	0.03	1000000	6									
	10.1007/s11356-024-34659-y	693.9	69390000	*Aliivibrio fischeri*	EC50	30 min	69390	281.81	0.00	6939000	6.841297									
	10.1007/s11356-024-34659-y	17.1	1710000	*Escherichia coli*	EC50	24h	1710	281.81	0.16	171000	5.232996									
	10.1007/s11356-024-34659-y	2.11	211000	*Lemna minor*	EC50	7d	211	281.81	1.34	21100	4.324282									
	10.1007/s11356-024-34659-y	49.4	4940000	*Daphnia magna*	EC5	48h	4940	281.81	0.06	494000	5.693727									
	10.1016/j.aquatox.2014.08.015	0.00656	656	*Microcystis aeruginosa*	NOAEC	72h	0.656	281.81	429.59	656	2.816904									
	https://link.springer.com/article/10.1007/s10646-008-0209-x	123.1	12310000	*Daphnia magna*	EC50	48h	12310	281.81	0.02	1231000	6.090258									
	https://link.springer.com/article/10.1007/s10646-008-0209-x	70.4	7040000	*Moina macrocopa*	EC50	48h	7040	281.81	0.04	704000	5.847573									
	https://doi.org/10.1016/j.chemosphere.2004.07.017	1.53	153000	*Selenastrum capricornutum*	EC50	72h	153	281.81	1.84	15300	4.184691									
**SDZ**	ECOSAR	10.25	1025000	Green algae	EC50	96h	1025	53.31	0.05	102500	5.010724	5.916457	0.877334	9.01046	2	1.65684E-09	0.999999998	2.89112E-07		

	ECOSAR	1.88	188000	Daphinids	LC50	48h	188	53.31	0.28	18800	4.274158							
	ECOSAR	1516.1	151610000	Fish	LC50	96h	151610	53.31	0.00	15161000	7.180728							
	https://doi.org/10.1016/j.chemosphere.2009.02.002	212	21200000	*Daphnia magna*	EC50	48h	21200	53.31	0.00	2120000	6.326336							
	https://doi.org/10.1016/S0045-6535(99)00443-9	221	22100000	*Daphnia magna*	EC50	48h	22100	53.31	0.00	2210000	6.344392							
	https://doi.org/10.1016/j.chemosphere.2013.01.029	27.2	2720000	*Daphnia magna*	EC50	48h	2720	53.31	0.02	272000	5.434569							
	https://doi.org/10.1016/j.chemosphere.2013.01.029	188	18800000	*Daphnia magna*	EC50	48h	18800	53.31	0.00	1880000	6.274158							
	https://doi.org/10.1016/j.chemosphere.2013.01.029	310	31000000	*Daphnia magna*	EC50	48h	31000	53.31	0.00	3100000	6.491362							
	https://doi.org/10.1007/s00128-017-2153-z	81.6	8160000	*Scenedesmus obliquus*	EC50	96h	8160	53.31	0.01	816000	5.91169							
	ECOSAR	162.16	16216000	Green algae	EC50	96h	16216	130.12	0.01	1621.6	3.209944	1.510082	1.858668	86.16752	3	0.448109093	0.551890907	78.1931691
	ECOSAR	124.04	12404000	Daphinids	LC50	48h	12404	130.12	0.01	1240.4	3.093562							
	ECOSAR	1313.14	131314000	Fish	LC50	96h	131314	130.12	0.00	13131.4	4.118311							
	10.1016/j.aquatox.2014.08.015	0.0153	1530	*Microcystis aeruginosa*	EC10	72h	1.53	130.12	85.05	0.153	-0.815311							
	10.1016/j.aquatox.2014.08.015	0.1626	16260	*Microcystis aeruginosa*	EC50	72h	16.26	130.12	8.00	1.626	0.211121							
**CIP**	https://doi.org/10.1016/j.aquatox.2014.08.015	241	24100000	*Pseudomonas putida*	EC50	16min	24100	130.12	0.01	2410	3.382017							
	10.1016/j.chemosphere.2014.06.062	23	2300000	*Chlorella* sp	EC50	72h	2300	130.12	0.06	230	2.361728							
	10.1016/j.chemosphere.2014.06.062	71	7100000	*Moina macrocopa*	EC50	48h	7100	130.12	0.02	710	2.851258							
	https://doi.org/10.1590/s2175-97902019000217661	0.1724	17240	*Microcystis aeruginosa*	EC50	48h	17.24	130.12	7.55	1.724	0.236537							
	https://doi.org/10.1590/s2175-97902019000217661	0.1356	13560	*Microcystis panniformis*	EC50	48h	13.56	130.12	9.60	1.356	0.13226							
	https://doi.org/10.1002/etc.678	0.0102	1020	*Anabaena flos-aquae*	EC50	72h	1.02	130.12	127.57	0.102	-0.9914							

	https://doi.org/10.3390/jox14020042	0.01356	1356	*Microcystis panniformis*	EC50	96h	1.356	130.12	95.96	0.1356	-0.86774							
	https://doi.org/10.1093/jac/46.suppl_1.53	0.005	500	*Microcystis aeruginosa*	EC50	72h	0.5	130.12	260.24	0.05	-1.30103							
	https://doi.org/10.1016/j.ecoenv.2014.11.021	11.3	1130000	*Raphidocelis subcapitata*	EC50	72h	1130	130.12	0.12	113	2.053078							
	https://doi.org/10.1016/j.chemosphere.2014.02.003	4.45	445000	*Daphnia curvirostris*	EC50	48h	445	130.12	0.29	44.5	1.64836							
	10.1016/j.chemosphere.2020.127823	36	3600000	*Ceriodaphnia dubia*	EC50	48h	3600	130.12	0.04	360	2.556303							
	https://doi.org/10.1016/j.envpol.2021.116779	620	62000000	*Danio rerio*	EC50	66h	62000	130.12	0.00	6200	3.792392							
**ENR**	ECOSAR	561	56100000	Green algae	EC50	96h	56100	65.22	0.00	5610000	6.748963	5.602461	1.111821	11.64131	4	8.29204E-06	0.999991708	1.45E-03
	ECOSAR	505	50500000	Daphnids	LC50	48h	50500	65.22	0.00	5050000	6.703291							
	ECOSAR	4900	490000000	Fish	LC50	96h	490000	65.22	0.00	49000000	7.690196							
	10.1016/j.chemosphere.2014.06.062	111	11100000	*Chlorella* sp	EC50	72h	11100	65.22	0.01	1110000	6.045323							
	10.1016/j.chemosphere.2014.06.062	69	6900000	*Moina macrocopa*	EC50	48h	6900	65.22	0.01	690000	5.838849							
	https://link.springer.com/article/10.1007/s10646-008-0209-x	56.7	5670000	*Daphnia magna*	EC50	48h	5670	65.22	0.01	567000	5.753583							
	https://doi.org/10.1002/etc.678	0.173	17300	*Anabaena flos-aquae*	EC50	72h	17.3	65.22	3.77	1730	3.238046							
	https://doi.org/10.1897/04-210R.1	3.1	310000	*Raphidocelis subcapitata*	EC50	72h	310	65.22	0.21	31000	4.491362							
	https://doi.org/10.1002/wer.1631	7.9	790000	*Daphnia magna*	EC50	48h	790	65.22	0.08	79000	4.897627							
	https://doi.org/10.1016/j.etap.2019.103295	16.72	1672000	*Daphnia magna*	EC50	48h	1672	65.22	0.04	167200	5.223236							
	https://doi.org/10.1016/j.chemosphere.2014.02.003	16.34	1634000	*Daphnia magna*	EC50	48h	1634	65.22	0.04	163400	5.213252							
	https://doi.org/10.1016/j.chemosphere.2014.02.003	4.33	433000	*Daphnia curvirostris*	EC50	48h	433	65.22	0.15	43300	4.636488							

	10.1016/j.chemosphere.2020.127823	60	6000000	*Ceriodaphnia dubia*	EC 50	48h	6000	65.22	0.01	600000	5.778151							
	https://doi.org/10.1080/02757540.2021.1974007	150	15000000	*Danio rerio*	EC 50	96h	15000	65.22	0.00	1500000	6.176091							
	ECOSAR	43800	4380000000	Green algae	EC 50	96h	4380000	85.97	0.00	438000000	8.641474	6.230115	1.942209	13.7991	5	0.013490411	0.986509589	2.35402046
	ECOSAR	140000	14000000000	Daphnids	LC 50	48h	14000000	85.97	0.00	1400000000	9.146128							
	ECOSAR	309000	30900000000	Fish	LC 50	96h	30900000	85.97	0.00	3090000000	9.489958							
	https://doi.org/10.1038/srep40385	107.6	10760000	*Daphnia magna*	LC 50	96h	10760	85.97	0.01	1076000	6.031812							
	https://doi.org/10.1038/srep40385	175.8	17580000	*Daphnia magna*	LC 50	48h	17580	85.97	0.00	1758000	6.245019							
NOR	10.1007/s10646-009-0334-1	38.49	3849000	*Scenedesmus obliquus*	EC 50	48h	3849	85.97	0.02	384900	5.585348							
	https://doi.org/10.1016/j.chemosphere.2004.07.017	16.6	1660000	*Scenedesmus obliquus*	EC 50	72h	1660	85.97	0.05	166000	5.220108							
	https://doi.org/10.1016/j.chemosphere.2004.07.017	4.01	401000	*Scenedesmus obliquus*	NOEC	72h	401	85.97	0.21	401000	5.603144							
	https://doi.org/10.1016/j.chemosphere.2004.07.017	10.4	1040000	*Chlorella vugaris*	EC 50	72h	1040	85.97	0.08	104000	5.017033							
	https://doi.org/10.1016/j.chemosphere.2004.07.017	4.02	402000	*Chlorella vugaris*	NOEC	72h	402	85.97	0.21	402000	5.604226							
	https://doi.org/10.1016/j.watres.2013.01.020	5.6	560000	*Anabaena CPB 4337*	EC 50	72h	560	85.97	0.15	56000	4.748188							
	https://doi.org/10.1016/j.aquatox.2021.105826	0.03479	3479	*Microcystis aeruginosa*	EC 50	72h	3.479	85.97	24.71	347.9	2.541454							
	https://doi.org/10.1016/j.envpol.2021.116779	1311	1311000000	*Danio rerio*	EC 50	66h	131100	85.97	0.00	13110000	7.117603							
	10.1016/j.scitotenv.2020.139499	0.1	100000	*Pseudanabaena sp*	LC 50	**8d**	100	175.14	1.75	10000	4	4.726603	1.411127	37.0541	6	0.039225863	0.960774137	6.844749621
LEV	https://doi.org/10.1080/26395940.2022.2130825	0.4376	43760	*Microcystis aeruginosa*	EC 50	96h	43.76	175.14	4.00	4376	3.641077							
	https://doi.org/10.1016/j.watres.2013.01.020	4.8	480000	*Anabaena CPB 4337*	EC 50	72h	480	175.14	0.36	48000	4.681241							

	https://doi.org/10.1897/04-210R.1	7.4	740000	*Raphidocelis subcapitata*	EC50	72h	740	175.14	0.24	74000	4.869232							
	https://doi.org/10.1016/j.chemosphere.2020.127823	28	2800000	*Daphnia magna*	EC50	48h	2800	175.14	0.06	280000	5.447158							
	https://doi.org/10.1002/wer.1631	19.5	1950000	*Daphnia magna*	EC50	48h	1950	175.14	0.09	195000	5.290035							
	https://doi.org/10.1016/j.ecoenv.2021.112778	15.11	1511000	*Daphnia magna*	EC50	48h	1511	175.14	0.12	151100	5.179264							
	10.1016/j.chemosphere.2020.127823	35	3500000	*Ceriodaphnia dubia*	EC50	48h	3500	175.14	0.05	350000	5.544068							
	https://doi.org/10.1897/04-210R.1	0.0079	7900	*Microcystis aeruginosa*	EC50	**5d**	7.9	175.14	22.17	790	2.897627							
	https://doi.org/10.1897/04-210R.1	0.051	5100	*Lemna menor*	EC50	7d	5.1	175.14	34.34	510	2.70757							
	https://doi.org/10.1016/j.envpol.2021.116779	5437	543700000	*Danio rerio*	EC50	66h	543700	175.14	0.00	54370000	7.735359							
	ECOSAR	7.95	795000	Green algae	EC50	96h	795	415.25	0.52	79500	4.900367	5.4467555	1.088803	76.23805	7	0.004691738	0.995308262	0.818688644
	ECOSAR	4.44	444000	Daphnids	LC50	48h	444	415.25	0.94	44400	4.647383							
	ECOSAR	47.67	4767000	Fish	LC50	96h	4767	415.25	0.09	476700	5.678245							
	https://doi.org/10.1016/S0045-6535(99)00443-9	100	10000000	*Daphnia magna*	LOEC	48h	10000	415.25	0.04	1000000	7							
	https://doi.org/10.1016/j.etap.2013.07.019	127.6	12760000	*Danio rerio*	EC50	72h	12760	415.25	0.03	1276000	6.105851							
**OTC**	https://link.springer.com/article/10.1007/s10646-008-0209-x	621.2	62120000	*Daphnia magna*	EC50	48h	62120	415.25	0.01	6212000	6.793231							
	https://link.springer.com/article/10.1007/s10646-008-0209-x	126.7	12670000	*Moina macrocopa*	EC50	48h	12670	415.25	0.03	1267000	6.102777							
	https://link.springer.com/article/10.1007/s10646-008-0209-x	110.1	11010000	*Oryzias latipe*	LC50	96h	11010	415.25	0.04	1101000	6.041787							
	https://doi.org/10.1016/j.chemosphere.2004.07.017	0.342	34200	*Scenedesmus obliquus*	EC50	72h	34.2	415.25	12.14	3420	3.534026							
	https://doi.org/10.1016/j.chemosphere.2004.07.017	0.183	18300	*Scenedesmus obliquus*	NOEC	72h	18.3	415.25	22.69	18300	4.262451							
	https://doi.org/10.1016/j.chemosphere.2004.07.017	7.05	705000	*Chlorella vugaris*	EC50	72h	705	415.25	0.59	70500	4.848189							

**TC**	ECOSAR	3.3	330000	Green algae	EC50	96h	330	153.72	0.47	33000	4.518514	5.125078	1.03951	29.99369	8	0.002351771	0.997648229	0.410374301
	ECOSAR	2.87	287000	Daphnids	LC50	48h	287	153.72	0.54	28700	4.457882							
	ECOSAR	27.09	2709000	Fish	LC50	96h	2709	153.72	0.06	270900	5.432809							
	https://doi.org/10.1016/S0045-6535(99)00443-9	340	34000000	*Daphnia magna*	NOEC	48h	34000	153.72	0.00	34000000	7.531479							
	https://doi.org/10.1016/j.ecoenv.2019.02.063	7.73	773000	*Chlorella vulgaris*	EC50	96h	773	153.72	0.20	77300	4.888179							
	https://doi.org/10.3390/agronomy12102497	10	1000000	*Chlorella pyrenoidosa*	EC50	96h	1000	153.72	0.15	100000	5							
	https://www.researchgate.net/publication/314278553_Ecotoxicity_of_selected_antibiotics_for_organisms_of_aquatic_and_terrestrial_ecosystems	1.82	182000	*Pseudokirchneriella subcapitata*	EC50	72h	182	153.72	0.84	18200	4.260071							
	https://www.researchgate.net/publication/314278553_Ecotoxicity_of_selected_antibiotics_for_organisms_of_aquatic_and_terrestrial_ecosystems	8.16	816000	*Daphnia magna*	EC50	48h	816	153.72	0.19	81600	4.91169							
**AZI**	ECOSAR		2190000	Fish	LC50	96h	2190	145.84	0.07	219000	5.340444	3.955485	1.204023	36.87032	9	0.068372998	0.931627002	11.93080315
	ECOSAR		302000	Daphnids	LC50	48h	302	145.84	0.48	30200	4.480007							
	ECOSAR		187000	Green algae	EC50	96h	187	145.84	0.78	18700	4.271842							
	https://doi.org/10.1016/j.emcon.2019.08.004	226.66	22666000	*Vibrio fischeri*	EC50	15min	22666	145.84	0.01	2266600	6.355375							
	10.5281/zenodo.7991973	1.3	130000	*Daphnia magna*	EC50	48h	130	145.84	1.12	13000	4.113943							
	https://doi.org/10.1016/j.ecoenv.2021.112553	0.018	1800	*Raphidocelis subcapitata*	EC10	72h	1.8	145.84	81.02	900	2.954243							
	https://doi.org/10.1016/j.ecoenv.2021.112553	0.026	2600	*Raphidocelis subcapitata*	EC20	72h	2.6	145.84	56.09	650	2.812913							
	https://doi.org/10.1016/j.ecoenv.2021.112553	0.051	5100	*Raphidocelis subcapitata*	EC50	72h	5.1	145.84	28.60	510	2.707570							
	https://doi.org/10.1016/j.ecoenv.2021.112553	0.033	3300	*Raphidocelis subcapitata*	LOEC	72h	3.3	145.84	44.19	3300	3.518514							

	https://doi.org/10.1016/j.ecoenv.2021.112553	0.01	1000	*Raphidocelis subcapitata*	NOEC	72h	1	145.84	145.84	1000	3							
	ECOSAR	14.91	1491000	Green algae	EC50	96h	1491	260.86	0.17	149100	5.173478	5.820962	1.786024	44.8139	10	0.028310801	0.971689199	4.940116865
	ECOSAR	2.89	289000	Daphnids	LC50	48h	289	260.86	0.90	28900	4.460898							
	ECOSAR	37.02	3702000	Fish	LC50	96h	3702	260.86	0.07	370200	5.568436							
	https://doi.org/10.1016/j.etap.2013.07.019	132.4	13240000	*Danio rerio*	EC50	48h	13240	260.86	0.02	1324000	6.121888							
	https://doi.org/10.1016/j.ecoenv.2020.110207	25	2500000	*Raphidocelis subcapitata*	LOEC	96h	2500	260.86	0.10	2500000	6.39794							
**AMX**	10.5281/zenodo.7991973	10.3	1030000	*Daphnia magna*	EC50	48h	1030	260.86	0.25	103000	5.012837							
	https://doi.org/10.3390/toxics9080196	27.2	27200000	*Daphnia magna*	NOEC	**21 d**	27200	260.86	0.01	27200000	7.434569							
	https://doi.org/10.3390/toxics9080196	21.8	21800000	*Oryzias latipes*	NOEC	**40 d**	21800	260.86	0.01	21800000	7.338456							
	https://link.springer.com/article/10.1007/s10646-008-0209-x	3597	359700000	*Vibrio fischeri*	EC50	15min	359700	260.86	0.00	35970000	7.55594							
	https://link.springer.com/article/10.1007/s002449900435	0.0037	370	*Microcystis aeruginosa*	EC50	72h	0.37	260.86	705.03	37	1.568202							
	https://link.springer.com/article/10.1007/s002449900435	250	25000000	*Selenastrum capricornutum*	NOEC	72h	25000	260.86	0.01	25000000	7.39794							
	ECOSAR	14000	1400000000	Green algae	EC50	96h	1400000	358.52	0.00	140000000	8.146128	6.40746	2.727716	55.95353	11	0.078898866	0.921101134	13.76752336
	ECOSAR	8230	823000000	Daphnids	LC50	48h	823000	358.52	0.00	82300000	7.9154							
**MER**	ECOSAR	101000	10100000000	Fish	LC50	96h	101000000	358.52	0.00	10100000000	9.004321							
	https://doi.org/10.1016/j.envres.2024.119409	0.033	3300	*Lemna minor*	EC20	7 dias	3.3	350.80	106.30	825	2.916454							
	https://doi.org/10.1016/j.envres.2024.119409	1.135	113500	*Lemna minor*	EC50	7 dias	113.5	350.80	3.09	11350	4.054996							

Table S3 Occurrence of Antibiotic-Resistant Bacteria in Treated Effluent

Bacterial specie	β-lactam resistance phenotype	Others antimicrobial resistance	Month
Acinetobacter modestus	CR (no Carbapenemase detected)	CIP	Dec
Pseudomonas sp	TGC-R (No ESBL)	None	Dec
Pseudomonas sp		CIP	Dec
Aeromonas caviae	KPC	None	Nov- Mar
	ESBL	CIP	Nov- Mar
	CR (no Carbapenemase detected)	CIP	Dec; Feb
	TGC-R (no ESBL)		Mar
Aeromonas hydrophila	KPC	None	Dec
	ESBL	None	Feb; Dec
	TGC-R (no ESBL)	CIP	Feb;Mar
	CR (no Carbapenemase detected)	CIP	Dec
Aeromonas sp	ESBL	CIP	Dec
	CR (no Carbapenemase detected)	CIP	Dec
	TGC-R (no ESBL)	None	Dec
Citrobacter freundii complex	ESBL	None	Dec
		CIP, GEN, SMX	Nov
		CIP, SMX	Nov
	TGG-R (no ESBL)	CIP	Nov;Dec;Mar
Enterobacter cloacae complex	KPC	None	Dec; Feb
		CIP, AMI	Dec
		CIP, GEN, AMI	Dec
		CIP, GEN, AMI, SMX	Deb
	ESBL	CIP, GEN, SMX	Dec; Feb
		CIP, GEN, SMX	Nov
		SMX	Nov; Feb
	CR (no Carbapenemase detected)	CIP	Nov
	TGG-R (no ESBL)	CIP	Dec
		CIP, GEN, AMI, SMX	Feb
Escherichia coli	EPC (KPC)	CIP	Mar
		CIP, AMI	Mar
	ESBL	CIP	Dec;Feb; Mar
		CIP, GEN	Nov; Dec
		CIP, GEN, AMI, SMX	Nov
		CIP, GEN, SMX	Nov; Dec
		CIP, SMX	Nov; Dec; Mar
		SMX	Nov; Feb
	TGC-R (No ESBL)	SMX	Nov
Klebsiella aerogenes	TGC-R (No ESBL)	None	Mar
		GEN	Nov

Klebsiella oxytoca complex	ESBL	none	Dec
Klebsiella pneumoniae complex	KPC	None	Mar
		CIP	dec-mar
		CIP, GEN, AMI	Feb
		CIP, GEN, SMX	Nov
		CIP, SMX	Nov; Dec
	ESBL	CIP	Mar
		CIP, GEN	Dec; Mar
		CIP, GEN, AMI, SMX	Dec
		CIP, GEN, SMX	Nov; Feb
		CIP, SMX	Nov
		GEN, SMX	Nov
	CR (no Carbapenemase detected)	CIP, GEN, SMX	Feb
Kluyvera cryocrescens	ESBL	CIP, GEN, SMX	Nov
Providencia alcalifaciens	ESBL	SMX	Dec
Raoultella sp	NDM	none	Dec

TGC-R =Third generation Cephalosporin-resistant; CR = Carbapenem-resistant; KPC = Klebsiella pneumoniae carbapenemase, ESBL = Extended spectrum beta-lactamase, CIP = ciprofloxacin; SMX = sulfamethoxazole; GEN = gentamicin, AMI= amicacin.

Table S4. Linearity parameters for validation of the LC-MS/MS method.

Pharmaceutical	RT (min)	Precursor ion (m/z)	Production (m/z)	Fragmentor (V)	CE (V)	CAV (V)	Linearity (µg L-1)	R2	Intra-day RSD% (n=6)	LOD (ng L-1)	Matrix effect	Recovery (%)
**Fluoroquinolones**												
CIP	6.30	332.0	314.0*/231.0	110	20/35	4	1-20	0.996	9.64	0.400	52	81
ENR	6.10	360.1	294.1	120	20/35	4	1-20	0.986	6.54	0.400	41	76
NOR	5.96	320.0	302.0*/276.0	70	15/15	4	1-20	0.965	3.12	0.400	38	74
LEV	6.20	362.1	381.1	75	25	4	1-20	0.985	4.07	0.400	52	73
**Macrolide**												
AZI	14.33	749.5	749.4	120	40	10	1-20	0.987	9.65	0.020	28	76
**β-lactam**												
AMX	0.51	366.0	349.0*/207.7	105	4/8	7	1-20	0.984	8.63	0.140	45	82
**Carbapenem**												
MEP	4.70	383.0	319.1	100	35	8	1-20	0.988	10.25	0.120	51	80
**Sulfonamides**												
SMX	9.67	254.0	156.0*/108.0	120	11/25	4	1-20	0.981	10.12	0.008	51	78
SDZ	3.30	251.0	156.0*/108.0	100	11/21	4	1-20	0.978	14.74	0.008	38	77
**Tetracyclines**												
TC	6.26	445.0	409.9*/427.0	125	15/10	4	1-20	0.965	10.42	0.100	56	89
OTC	5.20	461.2	426.0*/443.0	130	20/10	4	1-20	0.945	5.87	0.200	51	82
**Aminoglycoside**												
GEN	0.50	478.3	450.3*/322.0	120	13	10	1-20	0.975	12.11	0.100	59	71

RT, Retention time; CAV, Cell accelerator voltage; CE, collision energy (water/sediment); LOD, Limit of detection. *Quantification ion. CIP, ciprofloxacin; ENR, enrofloxacin; NOR, norfloxacin; LEV, levofloxacin; AZI, azithromycin; AMX, amoxicillin; MEP, meropenem; SMX, sulfamethoxazole; SDZ, sulfadiazine; TC, tetracycline; OTC, oxytetracycline; GEN, gentamicin.

Table S5. Repeated-measures ANOVA for the effects of time (months) on physicochemical parameters of WWTP effluents.

Source of variation	pH	Conductivity (µS cm-1)	TSS (ppm)	A.C.	R.C.	DO (mg mL-1)
Time	20510.59***	8047.71***	1065.62***	100.55***	1734.00***	348.71**
D.F	1.32	1.05	2.29	1.00	1.45	1.11

A.C. = apparent color; DO= dissolved oxygen; R.C.= real color; D.F. Degrees of freedom: *,**,*** Significant at P<0.05, P<0.01, and P<0.001, respectively.

Table S6. Repeated-measures ANOVA for the effects of time (months) on antimicrobial concentrations in WWTP effluents.

Source of variation	SMX	SDZ	CIP	ENR	NOR	LEV	OCT	TC	AZI	AMX	MER	GEN
Time	54.96***	36.55**	68.04***	108.39***	13.42*	51.89***	20.90**	34.70**	9.57*	33.89**	11.86*	10.16*
D.F	1.73	1.68	1.48	2.06	1.48	1.94	1.83	1.43	1.99	1.21	1.38	1.18

D.F. Degrees of freedom: *,**,*** Significant at P<0.05, P<0.01, and P<0.001, respectively.

SMX, sulfamethoxazole; SDZ, sulfadiazine; CIP, ciprofloxacin; ENR, enrofloxacin; NOR, norfloxacin; LEV, levofloxacin; OTC, oxytetracycline; TC, tetracycline; DOX, doxycycline; AZI, azithromycin; AMX, amoxicillin; MEP, meropenem.
